# Assessment of Social Distancing for Controlling COVID-19 in Korea: An Age-Structured Modeling Approach

**DOI:** 10.3390/ijerph17207474

**Published:** 2020-10-14

**Authors:** Yongin Choi, James Slghee Kim, Heejin Choi, Hyojung Lee, Chang Hyeong Lee

**Affiliations:** 1 Department of Mathematical Sciences, Ulsan National Institute of Science and Technology, Ulsan 44919, Korea; yongin9@unist.ac.kr (Y.C.); jameskim@unist.ac.kr (J.S.K.); chlgmlwls@unist.ac.kr (H.C.); 2 Busan Center for Medical Mathematics, National Institute of Mathematical Sciences, Daejeon 34047, Korea

**Keywords:** COVID-19, mathematical modeling, age-structured model, social distancing, transmission rate, contact matrix

## Abstract

The outbreak of the novel coronavirus disease 2019 (COVID-19) occurred all over the world between 2019 and 2020. The first case of COVID-19 was reported in December 2019 in Wuhan, China. Since then, there have been more than 21 million incidences and 761 thousand casualties worldwide as of 16 August 2020. One of the epidemiological characteristics of COVID-19 is that its symptoms and fatality rates vary with the ages of the infected individuals. This study aims at assessing the impact of social distancing on the reduction of COVID-19 infected cases by constructing a mathematical model and using epidemiological data of incidences in Korea. We developed an age-structured mathematical model for describing the age-dependent dynamics of the spread of COVID-19 in Korea. We estimated the model parameters and computed the reproduction number using the actual epidemiological data reported from 1 February to 15 June 2020. We then divided the data into seven distinct periods depending on the intensity of social distancing implemented by the Korean government. By using a contact matrix to describe the contact patterns between ages, we investigated the potential effect of social distancing under various scenarios. We discovered that when the intensity of social distancing is reduced, the number of COVID-19 cases increases; the number of incidences among the age groups of people 60 and above increases significantly more than that of the age groups below the age of 60. This significant increase among the elderly groups poses a severe threat to public health because the incidence of severe cases and fatality rates of the elderly group are much higher than those of the younger groups. Therefore, it is necessary to maintain strict social distancing rules to reduce infected cases.

## 1. Introduction

Coronavirus Disease 2019 (COVID-19) is a novel viral disease that is currently threatening public health worldwide. The virus responsible for the disease was initially called Novel Coronavirus (2019-nCoV) due to its novelty. Analysis of the phylogeny and taxonomy of 2019-nCoV have shown that the virus belongs to the subgenus Sarbecovirus, which SARS-CoV belongs to [1], but is more closely related to bat SARS-CoV [2,3]. Thus, 2019-nCoV was named “severe acute respiratory syndrome coronavirus 2” or “SARS-CoV-2” [4]. Cases of SARS-CoV-2 display symptoms such as fever, dry cough, dyspnea, and diarrhea, which are similar to symptoms noted in MERS-CoV and SARS-CoV. However, the distribution of each symptom differs [5].

Since the first case was reported in Wuhan, China, in December 2019, the disease quickly spread to other countries around the world. On 11 March 2020, with over 110,000 confirmed cases and 4000 casualties from 114 countries, the World Health Organization (WHO) declared COVID-19 a pandemic [6,7]. On 16 August 2020, it was reported that more than 21 million people were infected with COVID-19, and 761 thousand casualties were recorded across the world [8]. There are currently multiple vaccine candidates on different platforms being developed to stop the spread of COVID-19 [9]. Multiple vaccine candidates such as those developed from Moderna and BioNTech have reached phase 3 clinical trials [10], but there is still no vaccine that has been approved for commercial/clinical usage. As there is currently no effective vaccine against the disease, nonpharmaceutical interventions, which include school closures, social distancing, and telecommuting, have been implemented to prevent the spread of COVID-19 [11]. In the case of Korea, as of 19 August 2020, there were more than 14,000 cases and 300 deaths since the first case of COVID-19 was reported in the country on 20 January [12].

It has been reported that the transmission rates of COVID-19 differ by age [13,14,15]. To capture the age-dependent transmission dynamics of COVID-19, age-structured modeling approaches have been used. An age-structured model fitted with epidemic data from China, Italy, Japan, Singapore, Canada, and Korea was used to investigate the effect of physical distancing measures on the reduction of the magnitude of the epidemic peak of COVID-19 [13]. A data-driven age-structured model was proposed to study the effect of nonpharmaceutical interventions on preventing the collapse of the health system in Brazil [14]. An age-structured mathematical model was developed to predict the epidemic size and investigate the impact of a full lockdown in USA, UAE, and Algeria [15].

In this work, we present an age-structured mathematical model for describing the age-dependent transmission dynamics of COVID-19. By using the epidemiological data in Korea, we estimate the transmission rate for each age group as a product of the infection probability and the element of the contact matrix for the age group. Our target area is Seoul city and Gyeonggi province, the most populated capital area in Korea [16]. One of the characteristics of the target area is that its proportion of young adults aged between 20 and 49 (44.9% in Seoul, 55.5% in Gyeonggi) who engage in active social activities is relatively higher than that in other areas [17]. In this study, we analyze the epidemiological data between 1 February and 15 June in the target area, as provided in [18,19]. The purpose of this study is to investigate the effect of the social distancing rule under various scenarios by using the age-structured model.

## 2. Materials and Methods

### 2.1. Epidemiological Data

**Figure 1:**
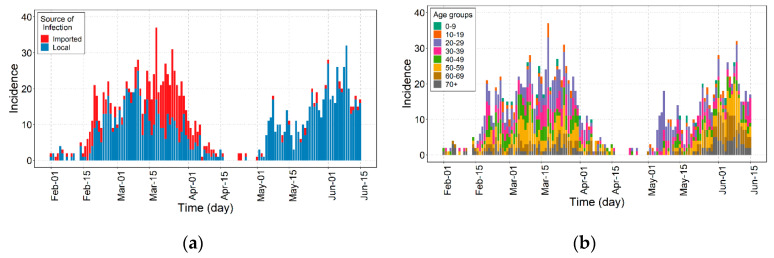
Epidemic curve of confirmed cases of COVID-19 in Seoul and Gyeonggi province, Korea by (**a**) source of infection for the imported (red) and local (blue) cases and (**b**) by age group.

In this study, we use the outbreak data of COVID-19 in the Seoul and Gyeonggi provinces between 1 February and 15 June 2020 [18,19]. Figure 1 shows the epidemic curve of confirmed cases of COVID-19 over the date of illness onset. A total of 1577 COVID-19 cases were reported. COVID-19 incidences were divided into different age groups to capture the age-dependent transmission dynamics. Figure 1a shows that the number of imported cases comprised about 39.6% infected cases before May but drastically diminished to about 4.0% afterward. Figure 1b shows that about 55.2% of infected cases were among ages 20–49 throughout the whole outbreak, and from May 1 through 14, about 76.8% of infected cases were among ages 20–39.

Table 1 shows the incidence data by age group and the sources of infection in the target area during the period. The sample dataset used in this study is shown in Table S1 in Supplementary Section A.

### 2.2. Timeline of Control Interventions

The transmission dynamics of COVID-19 are greatly affected by governmental control policies such as social distancing, school closures, and lockdowns. The Korean government has attempted to implement appropriate control policies in response to changes in the number of infected people. In Korea, on 23 February, the increasing level of COVID-19 cases raised the alert to its highest level of “Red”, thus strengthening the overall response system to possible epidemics [20]. As a result of this increase in the number of infected people, different levels of social distancing were implemented by the Korean government [21]. A brief description of the four levels of social distancing in Korea is shown in Table 2, and further details about the social distancing policies are given in Table S3 in Supplementary Section B.

**Figure 2:**
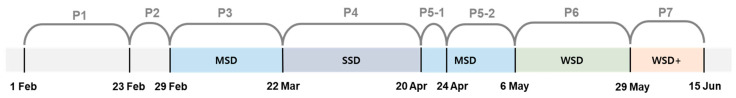
Timeline of social distancing and control interventions. Periods 1–7 are denoted by P1–P7, respectively.

Figure 2 shows the timeline of the governmental interventions with a focus on social distancing between 1 February and 15 June. Periods 1–7 are 1–22 February, 23–28 February, 29 February–21 March, 22 March–19 April, 20 April–5 May, 6–28 May, and 29 May–15 June. In particular, period 5 is divided into two subperiods around 24 April, because significant local transmission was presumed to begin on this date [23].

### 2.3. Contact Matrix

As Seoul and Gyeonggi provinces are densely populated with diverse people, to enhance the realism of our model, it is beneficial to consider the heterogeneity in contact networks. Two of the most important heterogeneous aspects of a contact network are location and age since different locations are often visited by certain age groups, which leads to consistent contact with specific age groups. For instance, people tend to have contact with people of a similar age outside their households (i.e., schools and workplaces). Since our age-structured model allows us to adjust the transmission rates among different age groups and since the location is closely linked to an individual’s contact pattern with certain age groups, we applied these location-based contact patterns to the transmission rates.

We divided the contact locations into four categories: school, workplace, household, and other locations. For each location category, we used the specific contact matrix of Korea from [21] to build our model. Each contact was defined by either physical or nonphysical contact; physical contact includes skin-to-skin contact like kissing, handshaking, etc., whereas nonphysical contact includes, e.g., a two-way conversation with three or more words in the physical presence of another person but no skin-to-skin contact [24].

Each location-specific contact matrix is a 16 × 16 square matrix, which represents the mean number of instances of contact between individuals of five-year age groups, such as 0–4, 5–9, 10–14, 15–19, 20–24, 25–29, 30–34, 35–39, 40–44, 45–49, 50–54, 55–59, 60–64, 65–69, 70–74, and 75 and above. Each element is the contact rate of an individual in one of the 16 age groups with people in the other 16 age groups at the specific locations. More precisely, the location-specific contact matrix M is written as [25]

(1) $$M=\left(m_{ij}\right),$$

where each element $m_{ij}$ denotes the mean number of contacts an individual in age group *i* makes with individuals in age group *j* per day. Note that contact matrix M is not necessarily symmetrical, which is a general feature that is also found in [26,27,28].

Since the focus areas are Seoul and Gyeonggi province, and the location-specific matrices of the whole region of Korea are only available in [25], we estimated the location-specific matrices of the focus area by using the proportion of the population of the area compared to that of Korea. We assumed the total population to be constant since the period of interest covers less than a year. We used the census data of Korea from January 2020 throughout the simulations. A summary of the data can be found in Figure S2 and Table S2 in Supplementary Section B, which describe how to calculate the contact matrix of the focus area. The calculated location-specific matrices for Seoul and Gyeonggi province are shown in Figure S4 in Supplementary Section B.

**Figure 3:**
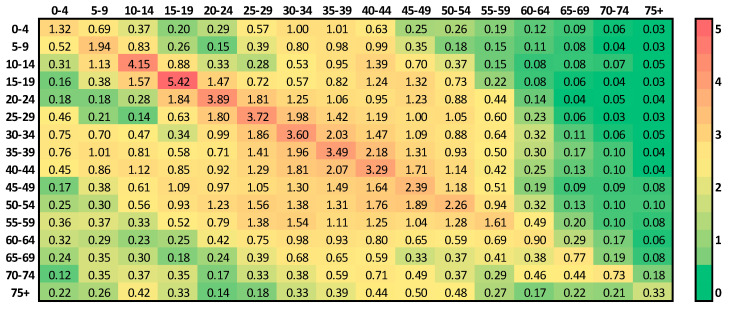
Contact matrix for a policy of school closure and no social distancing in Seoul and Gyeonggi province.

A full contact matrix M is composed of a linear combination of the location-specific contact matrices [25]:

(2) $$M=c_{W}\cdot m_{W}+c_{S}\cdot m_{S}+c_{H}\cdot m_{H}+c_{O}\cdot m_{O}.$$

where $m_{W}$ is the workplace contact matrix, $m_{S}$ is the school contact matrix, $m_{H}$ is the household contact matrix, and $m_{O}$ is the contact matrix for all other locations, except for the workplace, school, and household; $c_{W}$, $c_{S}$, and $c_{O}$ are constants, and $c_{H}$ is a 16 × 16 diagonal matrix, which are each multiplied by their respective matrices. Based on the real policies of school closure and social distancing levels in Korea, we composed five different contact matrices by adjusting $c_{W}$, $c_{S}$, $c_{H}$, and $c_{O}$ as $M^{O}$**,** $M^{C}$, $M_{w}^{C},$ $M_{m}^{C}$, and $M_{s}^{C}$, which denote the contact matrices of the cases of school openings with no social distancing, school closures with no social distancing, school closures with weak social distancing, school closures with medium social distancing, and school closures with strong social distancing, respectively. When the school is closed, $c_{S}=0$ since there are no contacts made in the school. On the other hand, when the school is closed, $c_{H}=diag{\left(1.5,\;1.5,\;1.5,\;1.5,\;1.1,\;1.1,\ldots ,\;1.1\right)}_{16}$, where $diag{\left(\;\right)}_{n}$ denotes the diagonal matrix with n diagonal entries, such that for age groups below the age of 20, contact rates increased by 50.0% and for age groups 20 and above, contact rates increased by 10.0% [29]. For social distancing, when there is no social distancing, weak social distancing, medium social distancing, or strong social distancing, we assumed $c_{O}=1,\;0.7,\;0.5,\;0.3,$ respectively, such that $c_{O}$ decreases under stronger social distancing. Note that different types of $c_{O}$ levels were tested while decreasing the orders of $c_{O}$ for stronger social distancing, as shown in Figures S12 and S13 in Supplementary Section D, but we present only one case due to the lack of a significant difference in the fitting and simulation results. An example of a scenario/policy-specific contact matrix of Seoul and Gyeonggi province—school closure with no social distancing, $M^{C}$—is shown in Figure 3; a comparison with the equivalent version for Korea is provided in Figure S3 in Supplementary Section B. Table 3 shows a summary of the contact matrices for different policies. The contact matrices for each scenario/policy are shown in Figure S5.

### 2.4. Mathematical Modeling

**Figure 4:**
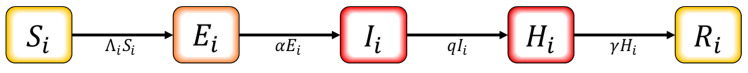
Schematic diagram for the mathematical model.

We developed a mathematical model to describe the transmission dynamics of COVID-19 by employing an *S–E–I–H–R* compartment model with 16 age groups. In this model, $S_{i},E_{i},I_{i},H_{i}$, and $R_{i}$ denote the susceptible, exposed, infectious, hospitalized, and recovered/removed population of age group i, respectively. The diagram for the model is shown in Figure 4.

The governing equation of the model is written as

(3) $$\begin{array}{l} \dot{S_{i}}=-\Lambda_{i}S_{i}\\ \dot{E_{i}}=\Lambda_{i}S_{i}-\alpha E_{i}\\ \dot{I_{i}}=\alpha E_{i}-qI_{i}\\ \dot{H_{i}}=qI_{i}-\gamma H_{i}\\ \dot{R_{i}}=\gamma H_{i} \end{array}$$

where $\Lambda_{i}=\underset{j}{\sum }\frac{\beta_{ji}I_{j}}{N_{j}}$ with $\beta_{ji}=m_{ij}b_{i}$ for i,j∈ {0–4, 5–9, 10–14, 15–19, 20–24, 25–29, 30–34, 35–39, 40–44, 45–49, 50–54, 55–59, 60–64, 65–69, 70–74, 75+}. The model parameters in Equation (3) are described in Table 4.

In this model, the asymptomatic infectious population is excluded. Although recent studies around the world suggest the presence and significance of an asymptomatic infectious population [31,32], we found that it is appropriate to apply the settings from our area of interest and the time period we are observing. Hence, we refer to a recent antibody test for COVID-19 for randomly selected subjects in Korea [33], which includes 1833 subjects from Seoul and 278 subjects from Gyeonggi province, where only 1 subject was found positive (A total of 3555 subjects were tested through a screening inspection and plague reduction neutralization test; 1555 serum samples were collected from 21 April through 19 June from 192 regions in Korea, and 1500 hospitalized patients from Seoul were tested from 25 May through 28 May). Thus, the ratio of asymptomatic infected people to infected people was estimated to be very small in Korea. For this reason, together with the difficulty in determining the proportion of asymptomatic infections accurately, we did not consider a compartment for asymptomatic infections in the mathematical model.

The parameter 1/q is the median value computed from the data for each period, and its values are given in Table 5. We estimate the transmission rate $\beta_{ij}$ by utilizing the least squares method, *lsqcurvefit*, which is an embedded function in MATLAB.

To measure the potential of the disease transmission in each period, we use the effective reproduction number $R_{t}$, which is the average number of secondary cases infected by an index case in a population of both susceptible and nonsusceptible hosts. $R_{t}$ is computed as $R_{t}=\rho \left(G\right),$ where ρ is the spectral radius of the next generation matrix G [34]. The derivation of the value of $R_{t}$ is described in Supplementary Section C.

### 2.5. Ethical Considerations

This study used the data available in [18,19]. The datasets were already fully anonymized and did not include any identity information. Thus, ethical approval was not required for this analysis.

### 2.6. Data Sharing Policy

The COVID-19 data for Gyeonggi province are accessible in [18], and the data for Seoul city are available upon request [19].

## 3. Result

### 3.1. Estimation of Transmission Rate

We estimated the transmission rate using the epidemiological data described in Section 2. Depending on each period, we estimated the transmission rates corresponding to the age group by applying the least squares method to the age-specific incidence data. We observed that the number of incidence data for each 5-year age group was not sufficient to estimate the transmission rate between age groups due to the absence of reported cases in some periods. Thus, to clarify the different properties of transmission rates between age groups, we estimated the transmission rate for 10-year age groups, such as 0–9, 10–19, 20–29, 30–39, 40–49, 50–59, 60–69, and 70 and above, by combining two 5-year age groups into one 10-year age group.

**Figure 5:**
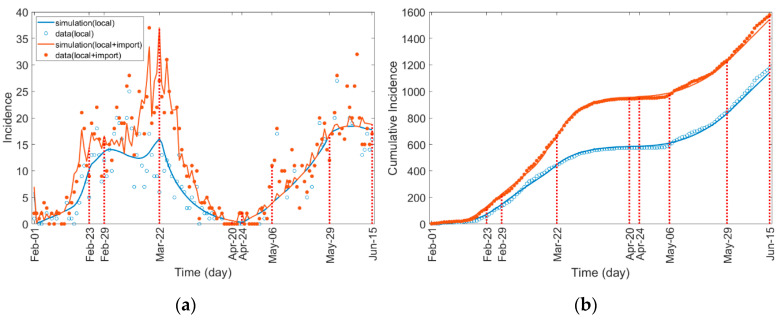
Incidence and cumulative incidence of all ages. Incidences by local transmission (local and imported transmission) are blue-colored (red-colored).

Figure 5 compares the observed and estimated COVID-19 cases for (a) incidence and (b) cumulative incidence among all ages. The results of the data-fitting for each age group are shown in Figures S6 and S7 in Supplementary Section D.

Table 5 shows the values of the estimated infection probability $\hat{b}$ and the effective reproduction number $R_{t}$ depending on the age group and period. Here, $\hat{b}$ is a vector consisting of the infection probability for eight age groups instead of sixteen age groups (i.e., $\hat{b}=\left\{{\hat{b}}_{k}\right\}$ for k∈{0–9, 10–19, 20–29, 30–39, 40–49, 50–59, 60–69, 70+}), and each subsequent pair of $b_{i}$ equals ${\hat{b}}_{k}$ (i.e., ${\hat{b}}_{0-9}=b_{0-4}=b_{5-9},$ ${\hat{b}}_{10-19}=b_{10-14}=b_{15-19},\cdots ,\;{\hat{b}}_{70+}=b_{70-74}=b_{75+}$). The value of $R_{t}$ was bigger than 2 in period 1, but after governmental control policies began in period 2, it decreased below 2. In particular, in periods 3 and 4, when medium and strong levels of social distancing were implemented, respectively, the value of $R_{t}$ became much less than 1. However, significant local infections have occurred since 24 April, when infected cases linked to club attendance among the young age groups were reported [23]. In the period between 24 April and 6 May, the $R_{t}$ value was estimated to be 2.4846, and the governmental control policies against local infections were implemented in period 7, which decreased the value of $R_{t}$ to 0.8047. In periods 1 and 2, the time taken to be diagnosed from symptom onset, *1*/*q*, was estimated at 8 and 5 days, respectively, but decreased to 3–4 days since 29 February when social distancing began. In the transition from period 2 to period 3, medium social distancing was implemented, and the infection probability $\hat{b}$ for age groups 0–9, 10–19, 20–29, 30–39, and 40–49 decreased by 37.8%, 86.1%, 21.3%, 40.6%, and 17.8%, respectively, while that of the age groups 50–59, 60–69, and 70+ increased by more than 400.0%, which resulted in a decrease of $R_{t}$ of 0.6776. Once the strong social distancing started in period 4, $\hat{b}$ either decreased or remained at similar level for almost all age groups, resulting in the $R_{t}$ decreasing by 0.1145. In period 5-2, the $\hat{b}$ for age groups 20–29 and 30–39 increased rapidly, resulting in an increase of $R_{t}$ of 2.4846.

### 3.2. Effect of the Control Strategies

We investigated the potential effect of social distancing under various scenarios. In Table 6, between 24 April and 31 August, we created seven scenarios along the baseline considering the social distancing strengths described in Section 2.2. These scenarios were designed to test the effects from the strongest case (scenario 1) to the weakest case (scenario 7).

**Figure 6:**
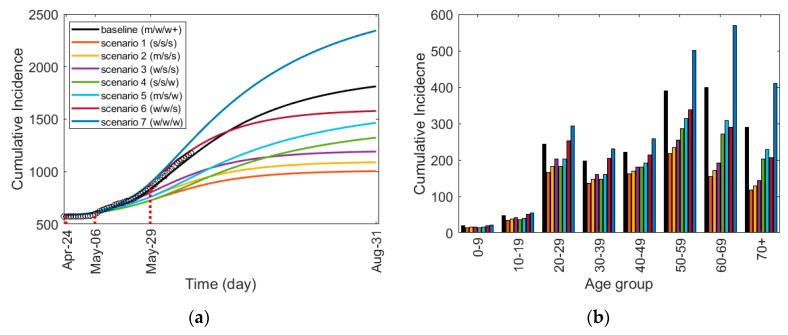
Cumulative Incidence for scenarios of social distancing: (**a**) the time-dependent cumulative incidence for the total age group and (**b**) the age-specific cumulative incidence from 1 February to 31 August 2020. Strong, medium, and weak social distancing is denoted by s, m, and w, respectively, and w+ denotes weak social distancing+ defined in Table 2.

Figure 6 shows (a) a comparison of the time-dependent cumulative incidences for the scenarios and (b) the age-specific cumulative incidences up to 31 August 2020. Figure 6a illustrates the effects of different social distancing combinations under each scenario, and in Figure 6b, we can observe how each scenario affects different age groups accordingly. The incidence plot corresponding to Figure 6a is shown in Figure S8a. The simulation results of the incidence and cumulative incidence for each age group are shown in Figures S9 and S10 in Supplementary Section D, respectively. Table 7 shows the cumulative incidence of each age group up to 31 August 2020.

Figure 6 and Table 7 show that if a strong level of social distancing had been maintained for all three periods, the number of infected people would have decreased by about 44.6%. On the other hand, if a weak level of social distancing was implemented for the three periods, the number of incidences would have increased by about 29.2%. However, when the intensity of social distancing is reduced in all the scenarios, the number of incidences increases in proportion with the degree of the intensity reduction. In particular, people who are between the ages of 0 and 19 present a minimum number of infected cases in all the scenarios of social distancing. In other words, the number of infected cases among those between the ages of 0 and 19 was the least affected by the strength of social distancing. For those between 50 and above, the number of infected cases increased drastically (28.4, 42.4, and 42.0 percent increase for age groups 50–59, 60–69, and 70+, respectively) in scenario 7 compared to the baseline, showing that without sufficiently strong social distancing, the age groups of 50 and above became noticeably vulnerable compared to the younger age groups. Scenarios 6 and 7 used the same weak social distancing strength from 24 April through 29 May. The only difference is in the social distancing strength during the longest period of 29 May–31 August, where scenario 6 uses strong social distancing, and scenario 7 uses weak social distancing. Despite the strength differences for the period of approximately three months, the effects on ages 0–19 appear to be minimal compared to those for people aged 20 and above. Moreover, the number of infected cases among those age 40 and above was effectively reduced even though social distancing was weak starting from 29 May. Corresponding to Figure 6b, the comparison of age for each scenario is shown in Figure S11a in Supplementary Section D.

**Figure 7:**
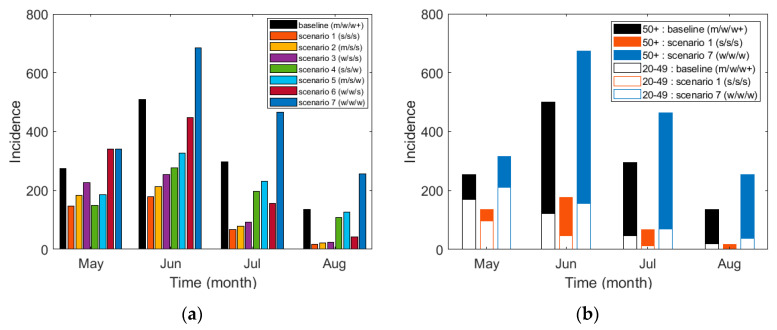
Cumulative incidence based on scenarios: (**a**) the monthly incidence for the total age group and (**b**) the monthly incidence of the two age groups of 20–49 and 50 and older (50+) for the baseline (scenarios 1 and 7).

Figure 7 shows (a) the monthly incidence of cases for the total age group under all scenarios and (b) a comparison of the monthly incidence among the two age groups of 20–49 and 50 and above for the baseline (scenarios 1 and 7). The monthly incidence of the other scenarios for these two age groups is shown in Figure S11b,c in Supplementary Section D. Table 8 shows the monthly incidence of the total age group, the age groups of 20–49, and those of 50 years and above for all scenarios. In the four months of May through August, compared to the baseline, the total incidence increased by 43.9% under scenario 7 but decreased by 66.6% for scenario 1.

## 4. Discussion

In this study, we analyzed the epidemiological data of COVID-19 cases in Seoul and Gyeonggi province between 1 February and 15 June 2020. The symptoms, transmission rates, and fatality rates of this disease differ by age, and the risks of severe symptoms and fatality rates are greater with an increase in age [13].

To take these aspects into account, we developed an age-structured model that describes the age-dependent dynamics of COVID-19. In the age-structured model developed in this study, we estimated the transmission rate by applying the contact matrix obtained from [25] to the actual incidence and population data for Seoul and Gyeonggi province. Since the control policies implemented by the governmental authorities affect the dynamics of infectious diseases [13,35], we divided the whole period between 1 February and 15 June into seven distinct periods following important changes in governmental control policies. We observed that the simulated incidence curve with the fitted transmission rate matches well with the actual incidence data of each age group over the whole period. Using the developed age-structured model, we investigated the effect of social distancing under various scenarios in the focus area.

For each of the seven distinct periods, we estimated the infection probability $\hat{b}$ for each age group and the effective reproduction number $R_{t}$, which led to three interesting results. First, as the social distancing strength increased, $R_{t}$ decreased from 2.1971 to 0.0001 until 24 April. Until the serious infections linked to clubs began to emerge, social distancing was effective in preventing local transmission. In period 5-2, the behavioral changes among those aged 20–39 [23] were suspected to be the primary cause of the escalating outbreaks after 24 April, among which the $R_{t}$ increased to 2.4846. Secondly, $\hat{b}$ differed greatly depending on the age group. Despite an increase in social distancing strength, the age groups 50 and above experienced an increase in $\hat{b}$ during period 3, while the transmission rates for the age groups younger than 50 decreased. This suggests that social distancing affects different age groups with different magnitudes, with younger age groups being more effective while under control. Thirdly, in period 6 during the weak social distancing, age groups 50 and above showed a greater change in $\hat{b}$ than age groups 20–49. This resulted in critical situations featuring an elevated number of deaths since the fatality rate is generally greater for people age 50 and above [17].

The baseline scenario reflected the actual social distancing policies implemented by the Korean governmental authorities between 1 February and 15 June 2020. In other scenarios, it was assumed that various levels of social distancing, different from the baseline scenario, were implemented in periods 5, 6, and 7. The simulation results in Table 7 showed that if a strong level of social distancing has been implemented for all three periods, the number of infected people would have decreased by about 44.6%. On the other hand, if a weak level of social distancing was maintained for the three periods, the number of infected people would have increased by about 29.2%. For all the scenarios, the results showed that a reduction in the intensity of social distancing produced an increase in the number of infected persons. Notably, the number of incidences in the age groups 60 years and above increased significantly compared to that of other age groups, which represents a very dangerous situation, as the fatality rate of the elderly groups is much higher than that of the younger groups [17]. Therefore, it is necessary to properly maintain a high-level intensity of social distancing to lower the fatality rate and reduce medical expenses. However, the social and economic costs that may emerge from strengthening social distancing should also be considered.

To investigate the effects of social distancing, we assumed that all schools were closed during the whole period of this study. We also reviewed some previous studies on the effects of school closures during different disease outbreaks [27,36]. Indeed, during the COVID-19 pandemic, many of our sampled schools have been opened since mid-May, except when there were recorded incidences of infected people in a school or its nearby area. Schools under such conditions were closed for a certain period, and quarantine policies were implemented differently for each school. However, it was difficult to provide an accurate reflection on the effects of schools opening/closing in this study since there are no reports on group infections in all schools over the whole period. Therefore, we propose that our model is more suitable for analyzing the impact of fixed and clear-cut control policies like social distancing, rather than the impact of schools opening/closing on the transmission of COVID-19.

Despite the limitations in our study, we successfully developed an age-structured model using the epidemiological data in Seoul and Gyeonggi province by implementing an age and location-based contact matrix, which is not a well-known model for COVID-19. Through this study, we analyzed the effects of different social distancing policies and further extended those effects to simulate different scenarios. As the social distancing strength was weakened, people age 50 and above were directly affected, showing a more significant increase in transmission rate than that among people age 20–49. Strong social distancing can be very effective in reducing the number of infected cases, as shown in scenario 1, where the cumulative incidence was reduced by 44.6% compared to the baseline.

## 5. Conclusions

In this paper, we developed an age-structured mathematical model for assessing the age-dependent transmission of COVID-19 in Korea. The target area was Seoul and Gyeonggi province, the most populated area in Korea. We divided the total human population in the target area into different age groups. We estimated the transmission rate for each age group in seven distinct periods using the COVID-19 data and contact matrix for each age group and investigated the effect of social distancing on the control of the disease in the age-structured model under various scenarios. In the most optimal scenario (Scenario 1), the reduced cumulative incidence of 44.6% from the baseline established that social distancing strength can have a critical impact on the mitigation of transmission dynamics.

Our modeling approach for COVID-19 has novelty in that we estimated the transmission rates of different age groups in seven distinct periods following government control policies. The modeling approach presented in this work can be applied to other target areas worldwide if sufficient epidemiological data and contact matrices for the various age groups are available.

## Figures and Tables

**Table 1:** Population summary of the cumulative incidence of COVID-19 by age group in Seoul and Gyeonggi province from 1 February 2020 through to 15 June 2020.

Age Group	Total	Source of Infection		Region	
		Local	Imported	Seoul	Gyeonggi
All age groups	1577 (100.0%)	1176 (74.6%)	401 (25.4%)	798 (50.6%)	779 (49.4%)
0–9	22 (1.4%)	19 (1.6%)	3 (0.7%)	7 (0.9%)	15 (1.9%)
10–19	61 (3.9%)	46 (3.9%)	15 (3.7%)	34 (4.3%)	27 (3.5%)
20–29	382 (24.2%)	224 (19.0%)	158 (39.4%)	215 (26.9%)	167 (21.4%)
30–39	271 (17.2%)	177 (15.1%)	94 (23.4%)	119 (14.9%)	152 (19.5%)
40–49	217 (13.8%)	172 (14.6%)	45 (11.2%)	104 (13.0%)	113 (14.5%)
50–59	279 (17.7%)	231 (19.6%)	48 (12.0%)	141 (17.7%)	138 (17.7%)
60–69	195 (12.4%)	170 (14.5%)	25 (6.2%)	101 (12.7%)	94 (12.1%)
70 and older	150 (9.5%)	137 (11.6%)	13 (3.2%)	77 (9.6%)	73 (9.4%)

**Table 2:** Description of the different levels of social distancing.

Social Distancing	Description
Weak Social Distancing (WSD)	Allows daily social and economic activities under epidemic prevention regulations while managing incidence levels under the capacity of the healthcare system. - Reduced school attendance (online lessons jointly implemented). - Public institutions operate with reduced density (one-third reduced).
Weak Social Distancing+ (WSD+)	While Weak Social Distancing is implemented, additional enhanced epidemic control measures are enforced [22]. - Most public institutions are controlled: events are canceled/postponed, facilities are closed, and work days/hours are reduced.
Medium Social Distancing (MSD)	Reduce incidence levels such that the healthcare system is able to function at its usual operating levels. - Large gatherings are strongly prohibited: limited social meetings/events (less than 50/100 attendees for indoor/outdoor), sporting events with no spectators on site, and the regulation of private/public facilities. - Limited school attendance (online lessons jointly implemented) with rotations by grade. Public institutions operate with reduced density (one-half reduced).
Strong Social Distancing (SSD)	Stop the rapid spread of disease and recover quarantine controls. - Any gatherings are strictly prohibited: no social meetings/events, no sporting events, and limited operations of all facilities. - School closing (online lessons or school closure). - Public institutions (corporates) are enforced (advised) with work-from-home protocols.

**Table 3:** Overview of the constants and contact matrixes used for each policy.

Policies	Notation	$c_{W}$	$c_{S}$	$c_{H}$ **	$c_{O}$
School Opening No Social Distancing	$M^{O}$	1	1	$I_{16}$	1
School Closing No Social Distancing	$M^{C}$	1	0 *	$diag{\left(1.5,\;1.5,\;1.5,\;1.5,\;1.1,\;1.1,\ldots ,\;1.1\right)}_{16}$	1
School Closing Weak Social Distancing	$M_{w}^{C}$	1 *	0 *	$diag{\left(1.5,\;1.5,\;1.5,\;1.5,\;1.1,\;1.1,\ldots ,\;1.1\right)}_{16}$	0.7 *
School Closing Weak Social Distancing+	$M_{w+}^{C}$	1 *	0 *	$diag{\left(1.5,\;1.5,\;1.5,\;1.5,\;1.1,\;1.1,\ldots ,\;1.1\right)}_{16}$	0.6 *
School Closing Medium Social Distancing	$M_{m}^{C}$	1 *	0 *	$diag{\left(1.5,\;1.5,\;1.5,\;1.5,\;1.1,\;1.1,\ldots ,\;1.1\right)}_{16}$	0.5 *
School Closing Strong Social Distancing	$M_{s}^{C}$	1 *	0 *	$diag{\left(1.5,\;1.5,\;1.5,\;1.5,\;1.1,\;1.1,\ldots ,\;1.1\right)}_{16}$	0.3 *

* Values with an asterisk (*) are assumed. ** *I*_16_ and *diag*(·)_16_ denote the 16 × 16 identity matrix and the diagonal matrix with diagonal entries, respectively.

**Table 4:** Descriptions of parameters.

Parameter	Description	Value	Reference
1/α	Incubation period (day)	5	[30]
1/q	Symptom onset to confirmed period (day)	Table 5	Estimated
$\beta_{ij}$	Transmission rate from age group i to j	Table 5	Estimated
$m_{ij}$	Number of contacts made by an individual in age group i with individuals in age group j	Figure 3	[25]
$b_{i}$	Infection probability of a person in age group i per contact	Table 5	Estimated
γ	Recovered/removed rate	*	[13,17]

* The recovered/removed rate varies by the age of the infected individual.

**Table 5:** Values of the infection probability for local transmission depending on age group and period.

Period	Time Interval	Contact Matrix	$\hat{b}$ *	$R_{t}$	1/q
P1	1 February–23 February	$M^{C}$	$2.95\times 10^{-4},\;5.58\times 10^{-3},\;1.93\times 10^{-3},\;1.75\times 10^{-2},$ $2.98\times 10^{-2},\;2.11\times 10^{-2},\;2.23\times 10^{-2},\;4.09\times 10^{-2}\;$	2.1971	8
P2	23 February–29 February	$M^{C}$	$9.04\times 10^{-3},\;2.02\times 10^{-2},\;1.74\times 10^{-2},\;1.87\times 10^{-2},$ $2.19\times 10^{-2},\;5.38\times 10^{-3},\;1.37\times 10^{-3},\;6.14\times 10^{-8}$	1.2173	5
P3	29 February–22 March	$M_{m}^{C}$	$5.62\times 10^{-3},\;2.78\times 10^{-3},\;1.37\times 10^{-2},\;1.11\times 10^{-2},$ $1.80\times 10^{-2},\;2.83\times 10^{-2},\;2.29\times 10^{-2},\;4.08\times 10^{-2}$	0.6776	4
P4	22 March–20 April	$M_{s}^{C}$	$6.73\times 10^{-11},\;2.86\times 10^{-3},\;7.83\times 10^{-8},\;2.93\times 10^{-3},$ $4.14\times 10^{-3},\;7.29\times 10^{-3},\;2.41\times 10^{-2},\;1.75\times 10^{-2}\;\;\;$	0.1145	3
P5-1	20 April–24 April	$M_{m}^{C}$	$1.57\times 10^{-9},\;1.83\times 10^{-10},\;9.53\times 10^{-6},\;5.23\times 10^{-11},$ $4.26\times 10^{-12},\;3.14\times 10^{-13},\;2.43\times 10^{-8},\;4.30\times 10^{-7}\;$	0.0001	3
P5-2	24 April–6 May	$M_{m}^{C}$	$5.58\times 10^{-3},\;2.84\times 10^{-2},\;1.16\times 10^{-1},\;4.40\times 10^{-2},$ $2.59\times 10^{-2},\;2.99\times 10^{-3},\;2.57\times 10^{-8},\;2.13\times 10^{-8}$	2.4846	4
P6	6 May–29 May	$M_{w}^{C}$	$1.38\times 10^{-2},\;2.06\times 10^{-2},\;4.25\times 10^{-2},\;4.80\times 10^{-2},$ $2.81\times 10^{-2},\;3.86\times 10^{-2},\;5.62\times 10^{-2},\;6.12\times 10^{-2}$	1.3804	3
P7	29 May–15 June	$M_{w+}^{C}$	$1.57\times 10^{-3},\;3.03\times 10^{-3},\;1.76\times 10^{-2},\;1.01\times 10^{-2},$ $1.81\times 10^{-2},\;5.26\times 10^{-2},\;1.34\times 10^{-1},\;1.25\times 10^{-1}$	0.8047	3

* In each cell for the fitted $\hat{b}$, the values from left to right at the top (bottom) are for the age groups of 0–9, 10–19, 20–29, 30–39, 40–49, 50–59, 60–69, and 70 and above, respectively.

**Table 6:** Scenarios of social distancing for periods 5-2, 6, and 7.

Scenario	Time Interval		
	24 April–6 May	6 May–29 May	29 May–31 August
baseline	Medium	Weak	Weak+
1	Strong	Strong	Strong
2	Medium	Strong	Strong
3	Weak	Strong	Strong
4	Strong	Strong	Weak
5	Medium	Strong	Weak
6	Weak	Weak	Strong
7	Weak	Weak	Weak

**Table 7:** Cumulative incidence for each age group from 1 February to 31 August 2020.

Scenario	Age Groups								
	Total	0–9	10–19	20–29	30–39	40–49	50–59	60–69	70+
baseline	1809	19	48	244	197	222	390	400	289
1	1002	14	35	165	136	162	218	154	118
	−44.6% *	−25.4%	−26.7%	−32.5%	−30.7%	−27.1%	−44.2%	−61.4%	−59.0%
2	1086	15	38	182	147	170	234	171	129
	−40.0%	−20.7%	−21.2%	−25.5%	−25.0%	−23.4%	−40.0%	−57.1%	−55.2%
3	1189	16	41	202	161	180	254	192	143
	−34.3%	−15.0%	−14.5%	−17.1%	−18.2%	−19.0%	−34.9%	−52.0%	−50.6%
4	1320	14	37	182	147	180	286	271	203
	−27.0%	−22.4%	−23.3%	−25.4%	−25.3%	−18.8%	−26.8%	−32.2%	−29.6%
5	1460	15	40	202	160	191	314	309	229
	−19.3%	−17.1%	−17.1%	−17.1%	−18.7%	−13.7%	−19.5%	−22.7%	−20.6%
6	1575	20	51	252	205	214	338	289	206
	−12.9%	5.7%	6.1%	3.0%	4.1%	−3.3%	−13.4%	−27.7%	−28.7%
7	2338	21	55	294	230	258	501	569	410
	29.2%	12.9%	14.4%	20.0%	17.0%	16.5%	28.4%	42.4%	42.0%

* The percentage represents the percentage increase or decrease from the baseline.

**Table 8:** Monthly incidence of the total age group, the age groups of 20–49, and those 50 years and older (50+) for all scenarios. The percentage below incidence represents the percentage increase or decrease from the baseline.

Scenario	May to August			May			June			July		August			
	Total	20–49	50+	Total	20–49	50+	Total	20–49	50+	Total	20–49	50+	Total	20–49	50+
baseline	1185	357	81	254	170	84	500	120	380	294	45	249	135	20	115
1	396	157	239	136	96	40	175	47	128	66	11	55	16	2	14
	−66.6% *	−55.8%	−71.1%	−46.5%	−43.7%	−51.8%	−65.0%	−60.7%	−66.1%	−77.6%	−75.0%	−77.9%	−88.1%	−85.6%	−87.0%
2	476	193	283	169	120	49	208	56	152	77	13	64	20	3	17
	−59.8%	−45.8%	−65.7%	−33.5%	−29.5%	−40.7%	−58.4%	−53.1%	−59.9%	−73.8%	−70.9%	−74.2%	−85.2%	−83.2%	−84.9%
3	573	236	337	210	149	61	248	67	181	90	15	75	23	3	20
	−51.6%	−33.8%	−59.2%	−17.3%	−12.4%	−27.4%	−50.4%	−43.9%	−52.4%	−69.4%	−65.9%	−69.8%	−83.0%	−80.3%	−82.3%
4	713	204	509	137	96	41	271	62	209	195	29	166	107	15	92
	−39.8%	−42.9%	−38.5%	−46.1%	−43.6%	−51.1%	−45.8%	−48.3%	−44.9%	−33.7%	−34.9%	−33.1%	−20.7%	−22.3%	−20.2%
5	850	248	602	170	120	50	322	74	248	229	34	195	125	18	107
	−28.3%	−30.6%	−27.3%	−33.1%	−29.4%	−39.9%	−35.6%	−38.4%	−34.7%	−22.1%	−23.8%	−21.5%	−7.4%	−9.0%	−6.5%
6	946	364	582	313	211	102	437	120	317	153	26	127	40	6	34
	−20.2%	2.0%	−29.7%	23.2%	24.0%	21.7%	−12.6%	−0.6%	−16.4%	−48.0%	−42.4%	−48.7%	−70.4%	−66.8%	−70.1%
7	1705	475	1230	314	211	103	673	156	517	463	70	393	253	37	216
	43.9%	33.1%	48.5%	23.6%	24.2%	23.3%	34.6%	29.7%	36.0%	57.5%	53.0%	57.9%	87.4%	82.8%	87.9%

* The percentage represents the percentage increase or decrease from the baseline.

## Supplementary Materials

The following are available online at https://www.mdpi.com/1660-4601/17/20/7474/s1, Section A: Data Analysis (Table S1: Dataset Sample; Figure S1: Cumulative incidence of Seoul/Gyeonggi Province by (a) age group, (b) source of infection, and (c) region); Section B: Contact Matrix and Control Policy (Figure S2. Population of South Korea and Seoul/Gyeonggi Province in January 2020 by age groups; Table S2. Summary of the population of South Korea and Seoul and Gyeonggi Province; Figure S3. Original and revised contact matrix (school closure): (a) is for Korea and (b) is for Seoul/Gyeonggi Province; Table S3. Description of different levels of Social Distancing; Figure S4. Location-specific contact matrices: (a) school contact matrix $m_{s}$, (b) other places contact matrix $m_{o}$, (c) workplace contact matrix $m_{w}$, and (d) household contact matrix $m_{h}$ for Seoul and Gyeonggi province; Figure S5. Scenario specific contact matrices: (a) school closed and no social distancing, (b) school open and no social distancing, (c) school closed and weak social distancing, (d) school closed and weak social distancing+, (e) school closed and medium social distancing, and (f) school closed and strong social distancing); Section C: The Effective Reproduction Number $R_{t}$; Section D: Result Figures (Figure S6. Estimation of transmission rate: Incidence of each age groups. Incidences by local transmission (local and imported transmission) are blue-colored (red-colored); Figure S7. Estimation of transmission rate: Cumulative Incidence of each age groups. Incidences by local transmission (local and imported transmission) are blue-colored (red-colored); Figure S8. Scenario simulation: (a) Incidence and (b) Cumulative Incidence of all ages. s, m, w denote strong, medium, weak social distancing, respectively, and w+ denotes weak social distancing+. Black circles are actual incidence data; Figure S9. Scenario simulation: Incidence of each age groups; Figure S10. Scenario simulation: Cumulative Incidence of each age groups; Figure S11. Other figures. (a) is scenario specific analysis on 31 Aug. 2020., (b) is 20–49 and (c) is 50 or older age group. s is strong-, m is medium-, w is weak- and w+ is weak social distancing+; Figure S12. Monthly incidence of two age groups of 20–49 and 50 or older (50+) for the baseline, scenario 1 and 7 on different types $c_{O}$ levels for strong, medium and weak social distancing: (a)$\;c_{O}=0.3,\;0.6,\;0.9$, (b)$\;c_{O}=0.3,\;0.6,\;0.9$, (c)$\;c_{O}=0.2,\;0.5,\;0.8$, (d); Figure S13. Monthly incidence of two age groups of 20-49 and 50 or older (50+) on different three types $c_{O}$ levels for strong, medium and weak social distancing: (a),(b)$\;c_{O}=0.3,\;0.6,\;0.9$, (c),(d)$\;c_{O}=0.2,\;0.5,\;0.8$, (e),(f)$\;c_{O}=0.3,\;0.5,\;0.8$).
