# Supplementary material for: Assessment of Social Distancing for Controlling COVID-19 in Korea: An Age-Structured Modeling Approach
Source: Int J Environ Res Public Health. 2020 Oct 14;17(20):7474. doi: 10.3390/ijerph17207474 (PMC7602130; doi:10.3390/ijerph17207474)
Supplement: Supplementary file 1 [file ijerph-17-07474-s001.pdf]

# Supplementary Section A.

## Data Analysis.

**Table S1.** Dataset Sample.

| ID | Symptom_date | Confirm_date | Source | Age | age_group | Region | Sex |
|---|---|---|---|---|---|---|---|
| 1 | 2020-02-01 | 2020-02-02 | Imported | 42 | 9 | Gyeonggi | M |
| 2 | 2020-02-01 | 2020-02-20 | Local | 84 | 16 | Seoul | M |
| 3 | 2020-02-02 | 2020-02-21 | Local | 35 | 8 | Seoul | M |
| 4 | 2020-02-02 | 2020-02-22 | Local | 62 | 13 | Seoul | M |
| 5 | 2020-02-03 | 2020-02-06 | Imported | 58 | 12 | Seoul | F |
| | | | … | | | | |
| 1573 | 2020-06-15 | 2020-06-19 | Local | 66 | 14 | Seoul | F |
| 1574 | 2020-06-15 | 2020-06-20 | Local | 36 | 8 | Gyeonggi | M |
| 1575 | 2020-06-15 | 2020-06-21 | Local | 38 | 8 | Seoul | M |
| 1576 | 2020-06-15 | 2020-06-23 | Imported | 57 | 12 | Seoul | M |
| 1577 | 2020-06-15 | 2020-06-28 | Local | 61 | 13 | Gyeonggi | F |

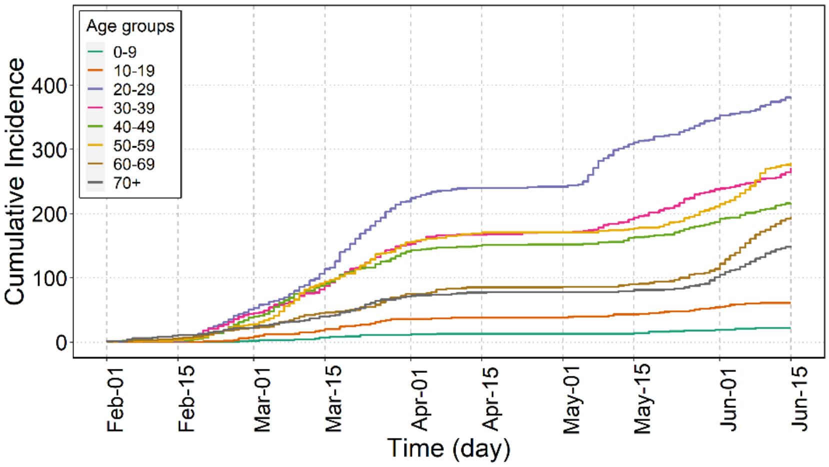


(a)

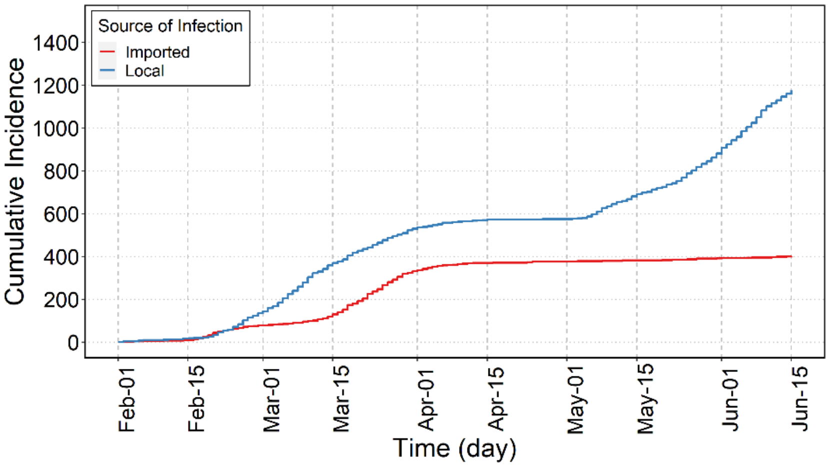


(b)

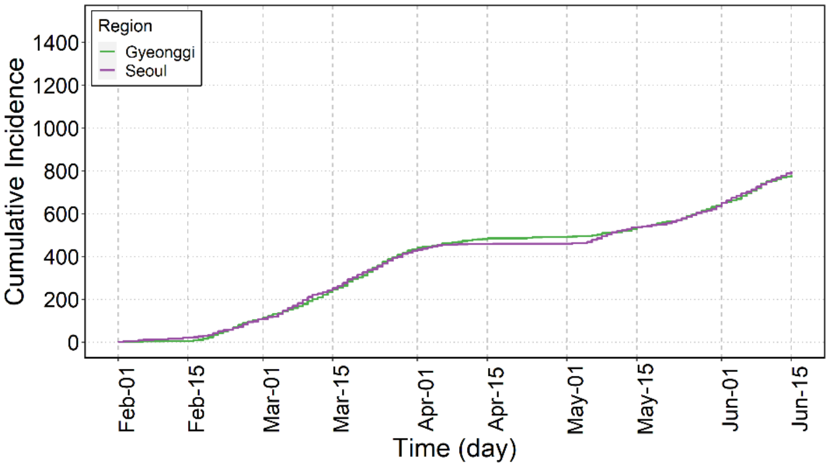


(c)

**Figure S1.** Cumulative incidence of Seoul/Gyeonggi Province by (a) age group, (b) source of infection, and (c) region.

# Supplementary Section B.

## Contact Matrix and Control Policy.

### The calculation of contact matrix in Seoul and Gyeonggi province

As stated in the main article, the definition of location-specific contact matrix $m_L$ is written as:

$$m_L = (m_{ij}),$$

where each element is $m_{ij}$, the mean number of contacts an individual in age group $i$ make with individuals in age group $j$ per day. More specifically, $m_{ij}$ can be defined as

$$m_{ij} = t_{ij}/n_i,$$

where $t_{ij}$ is the total number of contacts between individuals in age group $i$ and age group $j$ per day and $n_i$ is the total number of individuals in $i$, which can be found in Supporting Information of [26] .

By [24], we were given the location-specific contact matrix for school, workplace, household, and other locations (=all places excluding school, workplace, household) of South Korea. In [24], each element $m_{ij}$ of each location-specific matrix is estimated from measured empirical data of other countries for South Korea. These empirical data were collected through surveys in few countries to create a contact matrix, then using multiple data sources, these matrices were projected to fit the contact pattern of South Korea [24]. Though the location specific matrices were provided, the specific components $t_{ij}$ and $n_i$ were not provided. Since the population proportion for each age group differs between South Korea and Seoul and Gyeonggi Province, we consider the population proportion for each age group in both regions for modifying the location-specific contact matrices. The comparison of population between South Korea and Seoul/Gyeonggi Province by age groups is shown in Figure S3.

In order to obtain suitable contact matrices for Seoul and Gyeonggi Province, we first calculate the population ratio $z_j^{KR}$ of each age group $j$ for South Korea by dividing the population of age group $j$ by the total population of South Korea. Similarly, we calculate the population ratio $z_j^{SG}$ of each age group $j$ for Seoul and Gyeonggi and there are details in Table 3.

$$z_j^{KR} = \frac{\text{Population of age group } j \text{ in South Korea}}{\text{Total population of South Korea}} = \frac{n_j^{KR}}{\sum_j n_j^{KR}}$$

$$z_j^{SG} = \frac{\text{Population of age group } j \text{ in Seoul and Gyeonggi province}}{\text{Total population of Seoul and Gyeonggi province}} = \frac{n_j^{SG}}{\sum_j n_j^{SG}},$$

The mean number of contacts is between different age groups should be affected by the population ratio of each age groups. Hence, the ratio of the mean number of contacts made by an individual in age group $i$ and with individuals in age group $j$ per day from Korea to Seoul and Gyeonggi province was assumed to be the same to the ratio of the population ratio of age group $j$ from Korea to Seoul and Gyeonggi province. Thus,

$$\frac{m_{ij}^{KR}}{m_{ij}^{SG}} = \frac{z_j^{KR}}{z_j^{SG}},$$

where $m_{ij}^{KR}$ denotes the mean number of contacts made by an individual in age group $i$ and with individuals in age group $j$ per day in Korea, and $m_{ij}^{SG}$ denotes the mean number of contacts made by an individual in age group $i$ and with individuals in age group $j$ per day in Seoul and Gyeonggi.

Finally, we define the ratio between the population ratios of Korea and Seoul and Gyeonggi province as follows:

$$z_j = \frac{z_j^{SG}}{z_j^{KR}}.$$

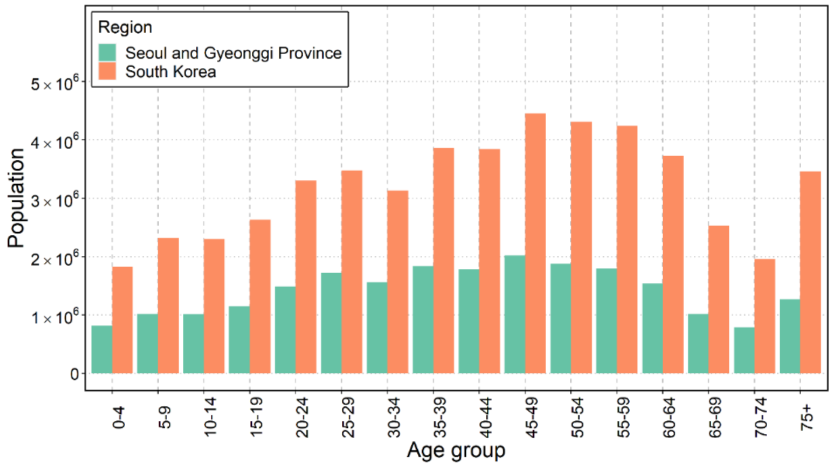


**Figure S2.** Population of South Korea and Seoul/Gyeonggi Province in January 2020 by age groups

**Table S2.** Summary of the population of South Korea and Seoul and Gyeonggi Province.

| Age group | Population of Korea | $z_j^{KR}$ | Population of Seoul and Gyeonggi | $z_j^{SG}$ | $z_j$ |
|---|---|---|---|---|---|
| All age groups | 51,348,927 | 1 | 22,701,328 | 1 | - |
| 0-4 | 1,827,170 | 0.0356 | 818,519 | 0.0361 | 1.0133 |
| 5-9 | 2,318,880 | 0.0452 | 1,020,669 | 0.0450 | 0.9956 |
| 10-14 | 2,298,127 | 0.0448 | 1,012,655 | 0.0446 | 0.9967 |
| 15-19 | 2,631,784 | 0.0513 | 1,148,471 | 0.0506 | 0.9871 |
| 20-24 | 3,302,097 | 0.0643 | 1,488,651 | 0.0656 | 1.0197 |
| 25-29 | 3,472,570 | 0.0676 | 1,721,853 | 0.0758 | 1.1216 |
| 30-34 | 3,128,471 | 0.0609 | 1,559,655 | 0.0687 | 1.1277 |
| 35-39 | 3,859,622 | 0.0752 | 1,837,053 | 0.0809 | 1.0766 |
| 40-44 | 3,842,664 | 0.0748 | 1,780,682 | 0.0784 | 1.0482 |
| 45-49 | 4,448,701 | 0.0866 | 2,022,509 | 0.0891 | 1.0283 |
| 50-54 | 4,308,633 | 0.0839 | 1,880,119 | 0.0828 | 0.9870 |
| 55-59 | 4,237,066 | 0.0825 | 1,794,019 | 0.0790 | 0.9577 |
| 60-64 | 3,721,669 | 0.0725 | 1,537,149 | 0.0677 | 0.9342 |
| 65-69 | 2,535,794 | 0.0494 | 1,017,605 | 0.0448 | 0.9077 |
| 70-74 | 1,958,693 | 0.0381 | 789,338 | 0.0348 | 0.9115 |
| 75+ | 3,456,986 | 0.0673 | 1272381 | 0.0560 | 0.8325 |

If $z > 1$, Seoul/Gyeonggi has a higher population proportion of that age group compared to that of South Korea. If $z < 1$, Seoul/Gyeonggi has a lower population proportion of that age group compared to that of South Korea.

Therefore $m_{ij}^{SG}$ can be made as follows:

$$m_{ij}^{SG} = m_{ij}^{KR} \cdot z_j.$$

| | 0-4 | 5-9 | 10-14 | 15-19 | 20-24 | 25-29 | 30-34 | 35-39 | 40-44 | 45-49 | 50-54 | 55-59 | 60-64 | 65-69 | 70-74 | 75+ |
|---|---|---|---|---|---|---|---|---|---|---|---|---|---|---|---|---|
| **0-4** | 1.30 | 0.69 | 0.37 | 0.20 | 0.28 | 0.51 | 0.88 | 0.93 | 0.60 | 0.24 | 0.26 | 0.20 | 0.13 | 0.10 | 0.06 | 0.04 |
| **5-9** | 0.51 | 1.95 | 0.83 | 0.27 | 0.15 | 0.34 | 0.71 | 0.91 | 0.94 | 0.34 | 0.18 | 0.16 | 0.12 | 0.09 | 0.04 | 0.04 |
| **10-14** | 0.30 | 1.14 | 4.16 | 0.89 | 0.33 | 0.25 | 0.47 | 0.88 | 1.33 | 0.68 | 0.37 | 0.15 | 0.08 | 0.09 | 0.08 | 0.07 |
| **15-19** | 0.16 | 0.38 | 1.58 | 5.49 | 1.44 | 0.64 | 0.51 | 0.76 | 1.18 | 1.29 | 0.74 | 0.23 | 0.09 | 0.06 | 0.04 | 0.03 |
| **20-24** | 0.17 | 0.18 | 0.28 | 1.86 | 3.82 | 1.61 | 1.11 | 0.98 | 0.91 | 1.19 | 0.89 | 0.46 | 0.15 | 0.05 | 0.06 | 0.05 |
| **25-29** | 0.45 | 0.21 | 0.14 | 0.63 | 1.77 | 3.32 | 1.76 | 1.31 | 1.13 | 0.98 | 1.06 | 0.63 | 0.25 | 0.07 | 0.03 | 0.03 |
| **30-34** | 0.74 | 0.70 | 0.47 | 0.35 | 0.97 | 1.66 | 3.19 | 1.89 | 1.41 | 1.06 | 0.89 | 0.67 | 0.34 | 0.12 | 0.07 | 0.06 |
| **35-39** | 0.75 | 1.02 | 0.81 | 0.58 | 0.69 | 1.26 | 1.74 | 3.24 | 2.08 | 1.27 | 0.94 | 0.52 | 0.32 | 0.19 | 0.11 | 0.04 |
| **40-44** | 0.44 | 0.87 | 1.13 | 0.86 | 0.90 | 1.15 | 1.61 | 1.92 | 3.14 | 1.66 | 1.16 | 0.44 | 0.26 | 0.15 | 0.11 | 0.05 |
| **45-49** | 0.16 | 0.38 | 0.61 | 1.10 | 0.95 | 0.94 | 1.15 | 1.38 | 1.56 | 2.33 | 1.19 | 0.53 | 0.21 | 0.10 | 0.09 | 0.10 |
| **50-54** | 0.25 | 0.30 | 0.57 | 0.94 | 1.21 | 1.39 | 1.22 | 1.22 | 1.68 | 1.83 | 2.29 | 0.98 | 0.34 | 0.14 | 0.10 | 0.11 |
| **55-59** | 0.35 | 0.37 | 0.33 | 0.52 | 0.78 | 1.23 | 1.36 | 1.03 | 1.19 | 1.02 | 1.29 | 1.68 | 0.53 | 0.22 | 0.11 | 0.09 |
| **60-64** | 0.32 | 0.30 | 0.23 | 0.26 | 0.41 | 0.67 | 0.87 | 0.86 | 0.76 | 0.63 | 0.60 | 0.72 | 0.96 | 0.32 | 0.19 | 0.07 |
| **65-69** | 0.24 | 0.35 | 0.30 | 0.19 | 0.24 | 0.35 | 0.61 | 0.60 | 0.56 | 0.32 | 0.37 | 0.43 | 0.41 | 0.85 | 0.21 | 0.09 |
| **70-74** | 0.12 | 0.35 | 0.37 | 0.36 | 0.17 | 0.29 | 0.34 | 0.55 | 0.68 | 0.48 | 0.38 | 0.30 | 0.49 | 0.49 | 0.80 | 0.22 |
| **75+** | 0.22 | 0.26 | 0.43 | 0.34 | 0.14 | 0.16 | 0.29 | 0.36 | 0.42 | 0.49 | 0.49 | 0.28 | 0.18 | 0.25 | 0.23 | 0.40 |

(a)

| | 0-4 | 5-9 | 10-14 | 15-19 | 20-24 | 25-29 | 30-34 | 35-39 | 40-44 | 45-49 | 50-54 | 55-59 | 60-64 | 65-69 | 70-74 | 75+ |
|---|---|---|---|---|---|---|---|---|---|---|---|---|---|---|---|---|
| **0-4** | 1.32 | 0.69 | 0.37 | 0.20 | 0.29 | 0.57 | 1.00 | 1.01 | 0.63 | 0.25 | 0.26 | 0.19 | 0.12 | 0.09 | 0.06 | 0.03 |
| **5-9** | 0.52 | 1.94 | 0.83 | 0.26 | 0.15 | 0.39 | 0.80 | 0.98 | 0.99 | 0.35 | 0.18 | 0.15 | 0.11 | 0.08 | 0.04 | 0.03 |
| **10-14** | 0.31 | 1.13 | 4.15 | 0.88 | 0.33 | 0.28 | 0.53 | 0.95 | 1.39 | 0.70 | 0.37 | 0.15 | 0.08 | 0.08 | 0.07 | 0.05 |
| **15-19** | 0.16 | 0.38 | 1.57 | 5.42 | 1.47 | 0.72 | 0.57 | 0.82 | 1.24 | 1.32 | 0.73 | 0.22 | 0.08 | 0.06 | 0.04 | 0.03 |
| **20-24** | 0.18 | 0.18 | 0.28 | 1.84 | 3.89 | 1.81 | 1.25 | 1.06 | 0.95 | 1.23 | 0.88 | 0.44 | 0.14 | 0.04 | 0.05 | 0.04 |
| **25-29** | 0.46 | 0.21 | 0.14 | 0.63 | 1.80 | 3.72 | 1.98 | 1.42 | 1.19 | 1.00 | 1.05 | 0.60 | 0.23 | 0.06 | 0.03 | 0.03 |
| **30-34** | 0.75 | 0.70 | 0.47 | 0.34 | 0.99 | 1.86 | 3.60 | 2.03 | 1.47 | 1.09 | 0.88 | 0.64 | 0.32 | 0.11 | 0.06 | 0.05 |
| **35-39** | 0.76 | 1.01 | 0.81 | 0.58 | 0.71 | 1.41 | 1.96 | 3.49 | 2.18 | 1.31 | 0.93 | 0.50 | 0.30 | 0.17 | 0.10 | 0.04 |
| **40-44** | 0.45 | 0.86 | 1.12 | 0.85 | 0.92 | 1.29 | 1.81 | 2.07 | 3.29 | 1.71 | 1.14 | 0.42 | 0.25 | 0.13 | 0.10 | 0.04 |
| **45-49** | 0.17 | 0.38 | 0.61 | 1.09 | 0.97 | 1.05 | 1.30 | 1.49 | 1.64 | 2.39 | 1.18 | 0.51 | 0.19 | 0.09 | 0.09 | 0.08 |
| **50-54** | 0.25 | 0.30 | 0.56 | 0.93 | 1.23 | 1.56 | 1.38 | 1.31 | 1.76 | 1.89 | 2.26 | 0.94 | 0.32 | 0.13 | 0.10 | 0.10 |
| **55-59** | 0.36 | 0.37 | 0.33 | 0.52 | 0.79 | 1.38 | 1.54 | 1.11 | 1.25 | 1.04 | 1.28 | 1.61 | 0.49 | 0.20 | 0.10 | 0.08 |
| **60-64** | 0.32 | 0.29 | 0.23 | 0.25 | 0.42 | 0.75 | 0.98 | 0.93 | 0.80 | 0.65 | 0.59 | 0.69 | 0.90 | 0.29 | 0.17 | 0.06 |
| **65-69** | 0.24 | 0.35 | 0.30 | 0.18 | 0.24 | 0.39 | 0.68 | 0.65 | 0.59 | 0.33 | 0.37 | 0.41 | 0.38 | 0.77 | 0.19 | 0.08 |
| **70-74** | 0.12 | 0.35 | 0.37 | 0.35 | 0.17 | 0.33 | 0.38 | 0.59 | 0.71 | 0.49 | 0.37 | 0.29 | 0.46 | 0.44 | 0.73 | 0.18 |
| **75+** | 0.22 | 0.26 | 0.42 | 0.33 | 0.14 | 0.18 | 0.33 | 0.39 | 0.44 | 0.50 | 0.48 | 0.27 | 0.17 | 0.22 | 0.21 | 0.33 |

(b)

**Figure S3.** Original and revised contact matrix (school closure): (a) is for Korea and (b) is for Seoul/Gyeonggi Province.

**Table S3.** Description of different levels of Social Distancing.

| Policy Type | Description |
|---|---|

| | |
|---|---|
| Weak Social Distancing (Distancing in Daily Life) | **Goal:** Allow daily social and economic activities under epidemic prevention regulations while managing incidence levels under the capacity of healthcare system<br><br>**Date: 6 May, 2020 – 28 May, 2020**<br>- Social meetings' and sporting events' admissions are allowed.<br>- Visits to multi-purpose facilities are allowed (High risk facility visits require face masks and registration on entrance).<br>- Public facilities may open depending on risk levels and administrative orders.<br>- School attendance and online lessons are jointly implemented.<br>- Public institutions operate under the condition of reduced density (e.g. 1/3 reduced) for each division.<br>- Corporates are advised to operate under similar conditions. |
| Weak Social Distancing + (Distancing in Daily Life with strengthened control measure for the Seoul Metropolitan Region) | While Distancing in Daily Life is implemented additional enhanced epidemic control measures are carried out.<br><br>**Date: 29 May, 2020 – Present**<br>Additional measures:<br>- Advise businesses to refrain from operating. Operating businesses must follow epidemic prevention regulations.<br>- Regular inspections will be made, and non-compliance will result in charge or prohibition.<br>- All public facilities are suspended from operation.<br>- All events held by government or public institutions must be cancelled are postponed.<br>- Public institutions' and corporates' work days/hours are adjusted flexibly to minimize contact between workers.<br>- Visits to high risk facilities (i.e. medical institutions and sanatoriums) are prohibited while face masks and symptom surveillance are mandatory for staffs.<br>- Unnecessary visits, meetings, and social events are advised to be avoided. |
| Medium Social Distancing (Social Distancing) | **Goal:** Reduce incidence levels such that the healthcare system is capable to handle at usual operating levels.<br><br>**Date: 29 February, 2020 – 21 March, 2020 / 20 April, 2020 – 5 May, 2020**<br>- Prohibit all private and public social meetings and events with large groups (more than 50 attendees for indoor and 100 attendees for outdoor).<br>- Sporting events must be held with no spectators on site.<br>- Private facilities may be suspended from operating or enforced to follow epidemic preventive regulations depending on group infection risk levels.<br>- School attendance and online lessons are jointly implemented while minimizing crowd density is enforced by reduction of students (rotational attendance by grade).<br>- Public institutions operate under the condition of reduced density (e.g. 1/2 reduced) for each division.<br>- Corporates are advised to operate under similar conditions. |

| | |
|---|---|
| Strong Social Distancing (Enhanced Social Distancing) | **Goal:** Stop the rapid spread of disease and recover quarantine controls.<br><br>**Date: 22 March, 2020 – 19 April, 2020**<br>- All meetings and events with 10 or more attendees are prohibited.<br>- All sporting events are prohibited.<br>- Nonessential multi-purpose facilities operate under limited conditions or suspended.<br>- No school attendance is allowed (online lessons or school closure).<br>- Public institutions are enforced with work-from-home with exceptions.<br>- Corporates are advised to operate under similar conditions. |

| | 0-4 | 5-9 | 10-14 | 15-19 | 20-24 | 25-29 | 30-34 | 35-39 | 40-44 | 45-49 | 50-54 | 55-59 | 60-64 | 65-69 | 70-74 | 75+ |
|---|---|---|---|---|---|---|---|---|---|---|---|---|---|---|---|---|
| **0-4** | 0.33 | 0.04 | 0.01 | 0.01 | 0.01 | 0.02 | 0.05 | 0.03 | 0.01 | 0.02 | 0.01 | 0.01 | 0.00 | 0.00 | 0.00 | 0.00 |
| **5-9** | 0.05 | 2.99 | 0.21 | 0.03 | 0.02 | 0.06 | 0.09 | 0.08 | 0.09 | 0.06 | 0.05 | 0.01 | 0.01 | 0.00 | 0.00 | 0.00 |
| **10-14** | 0.00 | 0.81 | 5.94 | 0.20 | 0.01 | 0.06 | 0.07 | 0.10 | 0.12 | 0.09 | 0.06 | 0.03 | 0.01 | 0.00 | 0.00 | 0.00 |
| **15-19** | 0.00 | 0.04 | 2.03 | 7.55 | 0.05 | 0.07 | 0.09 | 0.12 | 0.11 | 0.13 | 0.07 | 0.04 | 0.01 | 0.00 | 0.00 | 0.00 |
| **20-24** | 0.01 | 0.01 | 0.01 | 0.50 | 0.22 | 0.03 | 0.02 | 0.03 | 0.02 | 0.02 | 0.01 | 0.01 | 0.00 | 0.00 | 0.00 | 0.00 |
| **25-29** | 0.01 | 0.07 | 0.03 | 0.15 | 0.16 | 0.13 | 0.02 | 0.03 | 0.04 | 0.03 | 0.01 | 0.01 | 0.00 | 0.00 | 0.00 | 0.00 |
| **30-34** | 0.01 | 0.29 | 0.23 | 0.16 | 0.03 | 0.08 | 0.08 | 0.05 | 0.06 | 0.03 | 0.03 | 0.00 | 0.01 | 0.00 | 0.00 | 0.00 |
| **35-39** | 0.02 | 0.17 | 0.15 | 0.08 | 0.01 | 0.05 | 0.08 | 0.06 | 0.07 | 0.03 | 0.00 | 0.01 | 0.00 | 0.00 | 0.00 | 0.00 |
| **40-44** | 0.01 | 0.11 | 0.11 | 0.46 | 0.01 | 0.03 | 0.03 | 0.04 | 0.09 | 0.03 | 0.04 | 0.01 | 0.01 | 0.00 | 0.00 | 0.00 |
| **45-49** | 0.07 | 0.24 | 0.19 | 0.74 | 0.00 | 0.03 | 0.07 | 0.06 | 0.06 | 0.04 | 0.05 | 0.02 | 0.00 | 0.00 | 0.00 | 0.00 |
| **50-54** | 0.02 | 0.42 | 0.65 | 0.74 | 0.00 | 0.02 | 0.06 | 0.05 | 0.07 | 0.10 | 0.05 | 0.02 | 0.01 | 0.00 | 0.00 | 0.00 |
| **55-59** | 0.06 | 0.36 | 0.38 | 0.46 | 0.01 | 0.07 | 0.03 | 0.05 | 0.06 | 0.04 | 0.04 | 0.04 | 0.01 | 0.00 | 0.00 | 0.00 |
| **60-64** | 0.04 | 0.07 | 0.04 | 0.21 | 0.01 | 0.00 | 0.02 | 0.06 | 0.01 | 0.02 | 0.02 | 0.01 | 0.02 | 0.01 | 0.00 | 0.00 |
| **65-69** | 0.00 | 0.03 | 0.01 | 0.00 | 0.00 | 0.00 | 0.02 | 0.01 | 0.01 | 0.01 | 0.00 | 0.02 | 0.01 | 0.02 | 0.01 | 0.00 |
| **70-74** | 0.00 | 0.00 | 0.00 | 0.01 | 0.00 | 0.00 | 0.00 | 0.01 | 0.01 | 0.02 | 0.03 | 0.02 | 0.01 | 0.01 | 0.01 | 0.02 |
| **75+** | 0.00 | 0.02 | 0.00 | 0.02 | 0.00 | 0.01 | 0.00 | 0.00 | 0.00 | 0.00 | 0.00 | 0.00 | 0.00 | 0.00 | 0.00 | 0.00 |

Scale: 8 / 5.3 / 2.6 / 0

(a)

| | 0-4 | 5-9 | 10-14 | 15-19 | 20-24 | 25-29 | 30-34 | 35-39 | 40-44 | 45-49 | 50-54 | 55-59 | 60-64 | 65-69 | 70-74 | 75+ |
|---|---|---|---|---|---|---|---|---|---|---|---|---|---|---|---|---|
| **0-4** | 0.40 | 0.17 | 0.11 | 0.09 | 0.16 | 0.27 | 0.39 | 0.32 | 0.23 | 0.16 | 0.20 | 0.15 | 0.09 | 0.08 | 0.05 | 0.02 |
| **5-9** | 0.19 | 0.73 | 0.34 | 0.11 | 0.09 | 0.20 | 0.30 | 0.32 | 0.29 | 0.14 | 0.11 | 0.11 | 0.08 | 0.06 | 0.03 | 0.02 |
| **10-14** | 0.08 | 0.43 | 1.92 | 0.34 | 0.22 | 0.21 | 0.30 | 0.35 | 0.43 | 0.26 | 0.18 | 0.10 | 0.06 | 0.06 | 0.05 | 0.04 |
| **15-19** | 0.05 | 0.16 | 0.93 | 3.14 | 0.74 | 0.40 | 0.31 | 0.36 | 0.39 | 0.33 | 0.16 | 0.07 | 0.04 | 0.04 | 0.03 | 0.02 |
| **20-24** | 0.07 | 0.08 | 0.16 | 1.17 | 1.84 | 0.83 | 0.60 | 0.39 | 0.33 | 0.36 | 0.23 | 0.14 | 0.05 | 0.04 | 0.04 | 0.03 |
| **25-29** | 0.12 | 0.06 | 0.07 | 0.31 | 0.85 | 1.25 | 0.87 | 0.56 | 0.41 | 0.35 | 0.31 | 0.15 | 0.05 | 0.04 | 0.03 | 0.01 |
| **30-34** | 0.15 | 0.10 | 0.20 | 0.18 | 0.47 | 0.79 | 1.22 | 0.84 | 0.57 | 0.42 | 0.44 | 0.29 | 0.11 | 0.08 | 0.05 | 0.04 |
| **35-39** | 0.13 | 0.16 | 0.15 | 0.13 | 0.30 | 0.60 | 0.92 | 1.08 | 0.81 | 0.50 | 0.36 | 0.25 | 0.19 | 0.13 | 0.09 | 0.03 |
| **40-44** | 0.10 | 0.13 | 0.27 | 0.19 | 0.34 | 0.46 | 0.78 | 0.82 | 0.96 | 0.56 | 0.40 | 0.17 | 0.14 | 0.10 | 0.08 | 0.03 |
| **45-49** | 0.03 | 0.05 | 0.08 | 0.18 | 0.24 | 0.34 | 0.51 | 0.55 | 0.57 | 0.56 | 0.45 | 0.19 | 0.11 | 0.08 | 0.08 | 0.05 |
| **50-54** | 0.05 | 0.10 | 0.14 | 0.24 | 0.42 | 0.62 | 0.56 | 0.52 | 0.64 | 0.68 | 0.56 | 0.40 | 0.21 | 0.12 | 0.08 | 0.05 |
| **55-59** | 0.07 | 0.06 | 0.08 | 0.11 | 0.26 | 0.51 | 0.64 | 0.52 | 0.54 | 0.37 | 0.57 | 0.47 | 0.26 | 0.15 | 0.09 | 0.04 |
| **60-64** | 0.04 | 0.04 | 0.05 | 0.08 | 0.17 | 0.30 | 0.39 | 0.45 | 0.42 | 0.32 | 0.31 | 0.34 | 0.25 | 0.19 | 0.15 | 0.05 |
| **65-69** | 0.04 | 0.05 | 0.04 | 0.05 | 0.14 | 0.26 | 0.40 | 0.34 | 0.35 | 0.27 | 0.31 | 0.30 | 0.25 | 0.22 | 0.13 | 0.06 |
| **70-74** | 0.02 | 0.03 | 0.06 | 0.13 | 0.12 | 0.20 | 0.25 | 0.34 | 0.43 | 0.31 | 0.26 | 0.24 | 0.34 | 0.31 | 0.31 | 0.10 |
| **75+** | 0.02 | 0.02 | 0.04 | 0.03 | 0.06 | 0.09 | 0.21 | 0.16 | 0.17 | 0.20 | 0.15 | 0.13 | 0.11 | 0.15 | 0.14 | 0.08 |

Scale: 3 / 2 / 1 / 0

(b)

| | 0-4 | 5-9 | 10-14 | 15-19 | 20-24 | 25-29 | 30-34 | 35-39 | 40-44 | 45-49 | 50-54 | 55-59 | 60-64 | 65-69 | 70-74 | 75+ |
|---|---|---|---|---|---|---|---|---|---|---|---|---|---|---|---|---|
| **0-4** | 0.00 | 0.00 | 0.00 | 0.00 | 0.00 | 0.00 | 0.00 | 0.00 | 0.00 | 0.00 | 0.00 | 0.00 | 0.00 | 0.00 | 0.00 | 0.00 |
| **5-9** | 0.00 | 0.00 | 0.00 | 0.00 | 0.00 | 0.00 | 0.00 | 0.00 | 0.00 | 0.00 | 0.00 | 0.00 | 0.00 | 0.00 | 0.00 | 0.00 |
| **10-14** | 0.00 | 0.00 | 0.00 | 0.00 | 0.00 | 0.00 | 0.01 | 0.00 | 0.01 | 0.01 | 0.00 | 0.00 | 0.00 | 0.00 | 0.00 | 0.00 |
| **15-19** | 0.00 | 0.00 | 0.01 | 0.29 | 0.35 | 0.22 | 0.20 | 0.19 | 0.21 | 0.16 | 0.10 | 0.05 | 0.01 | 0.00 | 0.00 | 0.00 |
| **20-24** | 0.00 | 0.00 | 0.01 | 0.23 | 0.67 | 0.70 | 0.58 | 0.64 | 0.49 | 0.38 | 0.29 | 0.14 | 0.06 | 0.00 | 0.00 | 0.00 |
| **25-29** | 0.00 | 0.00 | 0.01 | 0.20 | 0.63 | 1.17 | 0.83 | 0.82 | 0.76 | 0.52 | 0.42 | 0.20 | 0.08 | 0.00 | 0.00 | 0.00 |
| **30-34** | 0.00 | 0.00 | 0.01 | 0.11 | 0.43 | 0.78 | 1.02 | 0.90 | 0.81 | 0.65 | 0.39 | 0.23 | 0.07 | 0.00 | 0.00 | 0.00 |
| **35-39** | 0.00 | 0.00 | 0.01 | 0.22 | 0.38 | 0.78 | 0.83 | 1.15 | 1.12 | 0.76 | 0.55 | 0.22 | 0.05 | 0.00 | 0.00 | 0.00 |
| **40-44** | 0.00 | 0.00 | 0.01 | 0.14 | 0.46 | 0.79 | 0.93 | 1.00 | 1.22 | 0.97 | 0.69 | 0.24 | 0.08 | 0.00 | 0.00 | 0.00 |
| **45-49** | 0.00 | 0.00 | 0.01 | 0.18 | 0.31 | 0.59 | 0.76 | 0.84 | 0.87 | 0.83 | 0.53 | 0.28 | 0.06 | 0.00 | 0.00 | 0.00 |
| **50-54** | 0.00 | 0.00 | 0.02 | 0.14 | 0.26 | 0.61 | 0.70 | 0.73 | 1.00 | 0.94 | 0.71 | 0.35 | 0.08 | 0.00 | 0.00 | 0.00 |
| **55-59** | 0.00 | 0.00 | 0.03 | 0.10 | 0.19 | 0.39 | 0.54 | 0.51 | 0.65 | 0.51 | 0.46 | 0.30 | 0.08 | 0.00 | 0.00 | 0.00 |
| **60-64** | 0.00 | 0.00 | 0.01 | 0.02 | 0.11 | 0.21 | 0.23 | 0.27 | 0.29 | 0.28 | 0.21 | 0.17 | 0.04 | 0.00 | 0.00 | 0.00 |
| **65-69** | 0.00 | 0.00 | 0.00 | 0.00 | 0.00 | 0.00 | 0.00 | 0.00 | 0.00 | 0.00 | 0.00 | 0.00 | 0.00 | 0.00 | 0.00 | 0.00 |
| **70-74** | 0.00 | 0.00 | 0.00 | 0.00 | 0.00 | 0.00 | 0.00 | 0.00 | 0.00 | 0.00 | 0.00 | 0.00 | 0.00 | 0.00 | 0.00 | 0.00 |
| **75+** | 0.00 | 0.00 | 0.00 | 0.00 | 0.00 | 0.00 | 0.00 | 0.00 | 0.00 | 0.00 | 0.00 | 0.00 | 0.00 | 0.00 | 0.00 | 0.00 |

Scale: 1 / 0.6 / 0.3 / 0

(c)

| | 0-4 | 5-9 | 10-14 | 15-19 | 20-24 | 25-29 | 30-34 | 35-39 | 40-44 | 45-49 | 50-54 | 55-59 | 60-64 | 65-69 | 70-74 | 75+ |
|---|---|---|---|---|---|---|---|---|---|---|---|---|---|---|---|---|
| **0-4** | 0.62 | 0.34 | 0.17 | 0.07 | 0.09 | 0.20 | 0.41 | 0.46 | 0.26 | 0.06 | 0.04 | 0.03 | 0.02 | 0.01 | 0.00 | 0.01 |
| **5-9** | 0.22 | 0.80 | 0.32 | 0.10 | 0.04 | 0.12 | 0.33 | 0.44 | 0.46 | 0.14 | 0.04 | 0.03 | 0.02 | 0.01 | 0.00 | 0.01 |
| **10-14** | 0.15 | 0.47 | 1.48 | 0.36 | 0.07 | 0.05 | 0.14 | 0.40 | 0.63 | 0.29 | 0.12 | 0.03 | 0.01 | 0.01 | 0.02 | 0.01 |
| **15-19** | 0.07 | 0.15 | 0.43 | 1.33 | 0.25 | 0.07 | 0.04 | 0.18 | 0.42 | 0.55 | 0.32 | 0.07 | 0.02 | 0.01 | 0.01 | 0.01 |
| **20-24** | 0.10 | 0.08 | 0.10 | 0.40 | 1.26 | 0.25 | 0.06 | 0.02 | 0.12 | 0.44 | 0.32 | 0.15 | 0.03 | 0.01 | 0.01 | 0.01 |
| **25-29** | 0.31 | 0.13 | 0.05 | 0.10 | 0.29 | 1.19 | 0.25 | 0.03 | 0.02 | 0.12 | 0.28 | 0.23 | 0.09 | 0.02 | 0.00 | 0.01 |
| **30-34** | 0.55 | 0.54 | 0.23 | 0.05 | 0.08 | 0.26 | 1.24 | 0.26 | 0.09 | 0.02 | 0.05 | 0.11 | 0.12 | 0.03 | 0.01 | 0.01 |
| **35-39** | 0.56 | 0.78 | 0.59 | 0.20 | 0.03 | 0.04 | 0.20 | 1.15 | 0.22 | 0.04 | 0.02 | 0.02 | 0.05 | 0.03 | 0.01 | 0.01 |
| **40-44** | 0.31 | 0.66 | 0.77 | 0.47 | 0.11 | 0.04 | 0.10 | 0.23 | 1.01 | 0.17 | 0.05 | 0.01 | 0.03 | 0.03 | 0.01 | 0.01 |
| **45-49** | 0.12 | 0.30 | 0.47 | 0.66 | 0.38 | 0.11 | 0.03 | 0.09 | 0.18 | 0.91 | 0.18 | 0.04 | 0.01 | 0.01 | 0.01 | 0.03 |
| **50-54** | 0.18 | 0.18 | 0.37 | 0.50 | 0.50 | 0.30 | 0.11 | 0.05 | 0.11 | 0.24 | 0.90 | 0.17 | 0.03 | 0.01 | 0.01 | 0.04 |
| **55-59** | 0.27 | 0.28 | 0.20 | 0.28 | 0.31 | 0.44 | 0.32 | 0.07 | 0.05 | 0.15 | 0.22 | 0.77 | 0.14 | 0.04 | 0.01 | 0.03 |
| **60-64** | 0.26 | 0.23 | 0.15 | 0.14 | 0.12 | 0.22 | 0.33 | 0.19 | 0.08 | 0.04 | 0.07 | 0.17 | 0.55 | 0.09 | 0.02 | 0.01 |
| **65-69** | 0.19 | 0.27 | 0.24 | 0.12 | 0.09 | 0.12 | 0.26 | 0.28 | 0.21 | 0.06 | 0.05 | 0.10 | 0.12 | 0.50 | 0.06 | 0.01 |
| **70-74** | 0.10 | 0.28 | 0.28 | 0.20 | 0.05 | 0.12 | 0.12 | 0.23 | 0.26 | 0.16 | 0.10 | 0.05 | 0.11 | 0.12 | 0.38 | 0.08 |
| **75+** | 0.18 | 0.22 | 0.35 | 0.28 | 0.07 | 0.08 | 0.11 | 0.20 | 0.25 | 0.28 | 0.30 | 0.12 | 0.06 | 0.07 | 0.06 | 0.23 |

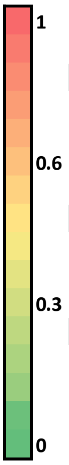


(d)

**Figure S4.** Location-specific contact matrices: (a) school contact matrix $m_s$, (b) other places contact matrix $m_o$, (c) workplace contact matrix $m_w$, and (d) household contact matrix $m_h$ for Seoul and Gyeonggi province.

| | 0-4 | 5-9 | 10-14 | 15-19 | 20-24 | 25-29 | 30-34 | 35-39 | 40-44 | 45-49 | 50-54 | 55-59 | 60-64 | 65-69 | 70-74 | 75+ |
|---|---|---|---|---|---|---|---|---|---|---|---|---|---|---|---|---|
| 0-4 | 1.32 | 0.69 | 0.37 | 0.20 | 0.29 | 0.57 | 1.00 | 1.01 | 0.63 | 0.25 | 0.26 | 0.19 | 0.12 | 0.09 | 0.06 | 0.03 |
| 5-9 | 0.52 | 1.94 | 0.83 | 0.26 | 0.15 | 0.39 | 0.80 | 0.98 | 0.99 | 0.35 | 0.18 | 0.15 | 0.11 | 0.08 | 0.04 | 0.03 |
| 10-14 | 0.31 | 1.13 | 4.15 | 0.88 | 0.33 | 0.28 | 0.53 | 0.95 | 1.39 | 0.70 | 0.37 | 0.15 | 0.08 | 0.08 | 0.07 | 0.05 |
| 15-19 | 0.16 | 0.38 | 1.57 | 5.42 | 1.47 | 0.72 | 0.57 | 0.82 | 1.24 | 1.32 | 0.73 | 0.22 | 0.08 | 0.06 | 0.04 | 0.03 |
| 20-24 | 0.18 | 0.18 | 0.28 | 1.84 | 3.89 | 1.81 | 1.25 | 1.06 | 0.95 | 1.23 | 0.88 | 0.44 | 0.14 | 0.04 | 0.05 | 0.04 |
| 25-29 | 0.46 | 0.21 | 0.14 | 0.63 | 1.80 | 3.72 | 1.98 | 1.42 | 1.19 | 1.00 | 1.05 | 0.60 | 0.23 | 0.06 | 0.03 | 0.03 |
| 30-34 | 0.75 | 0.70 | 0.47 | 0.34 | 0.99 | 1.86 | 3.60 | 2.03 | 1.47 | 1.09 | 0.88 | 0.64 | 0.32 | 0.11 | 0.06 | 0.05 |
| 35-39 | 0.76 | 1.01 | 0.81 | 0.58 | 0.71 | 1.41 | 1.96 | 3.49 | 2.18 | 1.31 | 0.93 | 0.50 | 0.30 | 0.17 | 0.10 | 0.04 |
| 40-44 | 0.45 | 0.86 | 1.12 | 0.85 | 0.92 | 1.29 | 1.81 | 2.07 | 3.29 | 1.71 | 1.14 | 0.42 | 0.25 | 0.13 | 0.10 | 0.04 |
| 45-49 | 0.17 | 0.38 | 0.61 | 1.09 | 0.97 | 1.05 | 1.30 | 1.49 | 1.64 | 2.39 | 1.18 | 0.51 | 0.19 | 0.09 | 0.09 | 0.08 |
| 50-54 | 0.25 | 0.30 | 0.56 | 0.93 | 1.23 | 1.56 | 1.38 | 1.31 | 1.76 | 1.89 | 2.26 | 0.94 | 0.32 | 0.13 | 0.10 | 0.10 |
| 55-59 | 0.36 | 0.37 | 0.33 | 0.52 | 0.79 | 1.38 | 1.54 | 1.11 | 1.25 | 1.04 | 1.28 | 1.61 | 0.49 | 0.20 | 0.10 | 0.08 |
| 60-64 | 0.32 | 0.29 | 0.23 | 0.25 | 0.42 | 0.75 | 0.98 | 0.93 | 0.80 | 0.65 | 0.59 | 0.69 | 0.90 | 0.29 | 0.17 | 0.06 |
| 65-69 | 0.24 | 0.35 | 0.30 | 0.18 | 0.24 | 0.39 | 0.68 | 0.65 | 0.59 | 0.33 | 0.37 | 0.41 | 0.38 | 0.77 | 0.19 | 0.08 |
| 70-74 | 0.12 | 0.35 | 0.37 | 0.35 | 0.17 | 0.33 | 0.38 | 0.59 | 0.71 | 0.49 | 0.37 | 0.29 | 0.46 | 0.44 | 0.73 | 0.18 |
| 75+ | 0.22 | 0.26 | 0.42 | 0.33 | 0.14 | 0.18 | 0.33 | 0.39 | 0.44 | 0.50 | 0.48 | 0.27 | 0.17 | 0.22 | 0.21 | 0.33 |

Colour scale: 0, 1, 2, 3, 4, 5

(a)

| | 0-4 | 5-9 | 10-14 | 15-19 | 20-24 | 25-29 | 30-34 | 35-39 | 40-44 | 45-49 | 50-54 | 55-59 | 60-64 | 65-69 | 70-74 | 75+ |
|---|---|---|---|---|---|---|---|---|---|---|---|---|---|---|---|---|
| 0-4 | 1.34 | 0.56 | 0.29 | 0.17 | 0.25 | 0.50 | 0.84 | 0.81 | 0.51 | 0.24 | 0.25 | 0.18 | 0.11 | 0.09 | 0.06 | 0.03 |
| 5-9 | 0.47 | 4.52 | 0.87 | 0.24 | 0.14 | 0.38 | 0.72 | 0.84 | 0.85 | 0.34 | 0.21 | 0.15 | 0.11 | 0.08 | 0.04 | 0.03 |
| 10-14 | 0.23 | 1.71 | 9.35 | 0.90 | 0.31 | 0.32 | 0.52 | 0.85 | 1.19 | 0.64 | 0.37 | 0.16 | 0.08 | 0.08 | 0.06 | 0.05 |
| 15-19 | 0.12 | 0.34 | 3.39 | 12.31 | 1.39 | 0.75 | 0.64 | 0.85 | 1.14 | 1.18 | 0.64 | 0.22 | 0.08 | 0.05 | 0.03 | 0.02 |
| 20-24 | 0.17 | 0.18 | 0.27 | 2.29 | 3.99 | 1.81 | 1.27 | 1.08 | 0.96 | 1.21 | 0.86 | 0.44 | 0.14 | 0.04 | 0.05 | 0.04 |
| 25-29 | 0.43 | 0.27 | 0.16 | 0.77 | 1.93 | 3.73 | 1.98 | 1.44 | 1.22 | 1.03 | 1.03 | 0.59 | 0.22 | 0.06 | 0.03 | 0.03 |
| 30-34 | 0.71 | 0.93 | 0.68 | 0.49 | 1.01 | 1.91 | 3.55 | 2.06 | 1.53 | 1.12 | 0.90 | 0.63 | 0.31 | 0.11 | 0.06 | 0.05 |
| 35-39 | 0.72 | 1.11 | 0.90 | 0.64 | 0.72 | 1.46 | 2.02 | 3.43 | 2.22 | 1.34 | 0.93 | 0.50 | 0.29 | 0.17 | 0.10 | 0.04 |
| 40-44 | 0.42 | 0.91 | 1.15 | 1.27 | 0.91 | 1.32 | 1.83 | 2.08 | 3.28 | 1.73 | 1.17 | 0.43 | 0.25 | 0.13 | 0.10 | 0.04 |
| 45-49 | 0.22 | 0.59 | 0.76 | 1.76 | 0.93 | 1.07 | 1.37 | 1.54 | 1.68 | 2.34 | 1.21 | 0.52 | 0.20 | 0.09 | 0.08 | 0.08 |
| 50-54 | 0.25 | 0.70 | 1.17 | 1.61 | 1.19 | 1.54 | 1.42 | 1.36 | 1.82 | 1.96 | 2.22 | 0.94 | 0.32 | 0.12 | 0.09 | 0.09 |
| 55-59 | 0.39 | 0.70 | 0.69 | 0.94 | 0.77 | 1.40 | 1.53 | 1.15 | 1.31 | 1.07 | 1.29 | 1.57 | 0.49 | 0.20 | 0.10 | 0.07 |
| 60-64 | 0.33 | 0.34 | 0.25 | 0.45 | 0.42 | 0.73 | 0.97 | 0.96 | 0.80 | 0.66 | 0.60 | 0.68 | 0.86 | 0.29 | 0.17 | 0.06 |
| 65-69 | 0.23 | 0.35 | 0.29 | 0.17 | 0.24 | 0.38 | 0.67 | 0.63 | 0.57 | 0.34 | 0.36 | 0.41 | 0.38 | 0.74 | 0.20 | 0.08 |
| 70-74 | 0.11 | 0.32 | 0.34 | 0.34 | 0.17 | 0.32 | 0.37 | 0.57 | 0.69 | 0.50 | 0.40 | 0.30 | 0.46 | 0.44 | 0.70 | 0.19 |
| 75+ | 0.20 | 0.26 | 0.39 | 0.32 | 0.13 | 0.18 | 0.32 | 0.37 | 0.41 | 0.48 | 0.45 | 0.26 | 0.16 | 0.22 | 0.20 | 0.31 |

Colour scale: 0, 4, 8, 12

(b)

| | 0-4 | 5-9 | 10-14 | 15-19 | 20-24 | 25-29 | 30-34 | 35-39 | 40-44 | 45-49 | 50-54 | 55-59 | 60-64 | 65-69 | 70-74 | 75+ |
|---|---|---|---|---|---|---|---|---|---|---|---|---|---|---|---|---|
| 0-4 | 1.20 | 0.64 | 0.33 | 0.17 | 0.24 | 0.49 | 0.88 | 0.91 | 0.56 | 0.20 | 0.20 | 0.14 | 0.10 | 0.07 | 0.04 | 0.03 |
| 5-9 | 0.46 | 1.72 | 0.72 | 0.23 | 0.12 | 0.33 | 0.71 | 0.88 | 0.90 | 0.31 | 0.15 | 0.12 | 0.08 | 0.06 | 0.03 | 0.03 |
| 10-14 | 0.28 | 1.01 | 3.57 | 0.78 | 0.27 | 0.22 | 0.44 | 0.85 | 1.26 | 0.62 | 0.31 | 0.12 | 0.06 | 0.06 | 0.06 | 0.04 |
| 15-19 | 0.14 | 0.33 | 1.29 | 4.47 | 1.24 | 0.60 | 0.48 | 0.71 | 1.12 | 1.22 | 0.68 | 0.20 | 0.07 | 0.04 | 0.03 | 0.02 |
| 20-24 | 0.16 | 0.15 | 0.23 | 1.49 | 3.34 | 1.56 | 1.07 | 0.94 | 0.85 | 1.12 | 0.81 | 0.40 | 0.12 | 0.03 | 0.04 | 0.03 |
| 25-29 | 0.42 | 0.19 | 0.12 | 0.53 | 1.55 | 3.35 | 1.72 | 1.25 | 1.06 | 0.90 | 0.95 | 0.55 | 0.21 | 0.05 | 0.02 | 0.02 |
| 30-34 | 0.71 | 0.67 | 0.41 | 0.29 | 0.84 | 1.63 | 3.23 | 1.78 | 1.30 | 0.96 | 0.75 | 0.56 | 0.29 | 0.09 | 0.05 | 0.04 |
| 35-39 | 0.72 | 0.96 | 0.76 | 0.54 | 0.62 | 1.24 | 1.69 | 3.17 | 1.93 | 1.16 | 0.82 | 0.42 | 0.24 | 0.13 | 0.07 | 0.03 |
| 40-44 | 0.42 | 0.82 | 1.04 | 0.79 | 0.82 | 1.15 | 1.58 | 1.82 | 3.00 | 1.54 | 1.02 | 0.37 | 0.20 | 0.10 | 0.07 | 0.03 |
| 45-49 | 0.16 | 0.37 | 0.59 | 1.04 | 0.90 | 0.95 | 1.15 | 1.32 | 1.47 | 2.23 | 1.04 | 0.45 | 0.16 | 0.06 | 0.06 | 0.07 |
| 50-54 | 0.24 | 0.27 | 0.52 | 0.85 | 1.11 | 1.37 | 1.21 | 1.15 | 1.57 | 1.68 | 2.09 | 0.82 | 0.25 | 0.09 | 0.07 | 0.08 |
| 55-59 | 0.34 | 0.35 | 0.30 | 0.49 | 0.72 | 1.23 | 1.34 | 0.95 | 1.09 | 0.93 | 1.11 | 1.47 | 0.42 | 0.15 | 0.07 | 0.06 |
| 60-64 | 0.31 | 0.28 | 0.21 | 0.23 | 0.36 | 0.66 | 0.86 | 0.79 | 0.67 | 0.55 | 0.50 | 0.59 | 0.82 | 0.23 | 0.13 | 0.05 |
| 65-69 | 0.23 | 0.33 | 0.29 | 0.17 | 0.20 | 0.31 | 0.56 | 0.54 | 0.48 | 0.25 | 0.27 | 0.32 | 0.31 | 0.70 | 0.16 | 0.06 |
| 70-74 | 0.12 | 0.34 | 0.35 | 0.31 | 0.14 | 0.27 | 0.31 | 0.49 | 0.58 | 0.40 | 0.29 | 0.22 | 0.36 | 0.35 | 0.64 | 0.15 |
| 75+ | 0.22 | 0.25 | 0.41 | 0.32 | 0.12 | 0.15 | 0.27 | 0.34 | 0.39 | 0.44 | 0.43 | 0.23 | 0.14 | 0.18 | 0.17 | 0.31 |

Colour scale: 0, 1, 2, 3, 4, 5

(c)

| | 0-4 | 5-9 | 10-14 | 15-19 | 20-24 | 25-29 | 30-34 | 35-39 | 40-44 | 45-49 | 50-54 | 55-59 | 60-64 | 65-69 | 70-74 | 75+ |
|---|---|---|---|---|---|---|---|---|---|---|---|---|---|---|---|---|
| **0-4** | 1.16 | 0.62 | 0.32 | 0.16 | 0.23 | 0.46 | 0.84 | 0.88 | 0.54 | 0.18 | 0.18 | 0.13 | 0.09 | 0.06 | 0.04 | 0.03 |
| **5-9** | 0.44 | 1.65 | 0.69 | 0.22 | 0.11 | 0.31 | 0.68 | 0.85 | 0.87 | 0.29 | 0.13 | 0.11 | 0.07 | 0.05 | 0.03 | 0.02 |
| **10-14** | 0.27 | 0.96 | 3.38 | 0.74 | 0.25 | 0.20 | 0.41 | 0.81 | 1.22 | 0.59 | 0.30 | 0.11 | 0.05 | 0.06 | 0.05 | 0.04 |
| **15-19** | 0.14 | 0.32 | 1.20 | 4.16 | 1.17 | 0.56 | 0.45 | 0.68 | 1.08 | 1.19 | 0.67 | 0.19 | 0.07 | 0.04 | 0.03 | 0.02 |
| **20-24** | 0.15 | 0.14 | 0.21 | 1.37 | 3.16 | 1.48 | 1.01 | 0.90 | 0.82 | 1.08 | 0.79 | 0.39 | 0.12 | 0.03 | 0.03 | 0.03 |
| **25-29** | 0.41 | 0.18 | 0.11 | 0.50 | 1.46 | 3.22 | 1.63 | 1.19 | 1.02 | 0.86 | 0.92 | 0.54 | 0.21 | 0.05 | 0.02 | 0.02 |
| **30-34** | 0.69 | 0.66 | 0.39 | 0.27 | 0.80 | 1.55 | 3.11 | 1.69 | 1.25 | 0.92 | 0.71 | 0.53 | 0.28 | 0.08 | 0.04 | 0.03 |
| **35-39** | 0.70 | 0.95 | 0.75 | 0.52 | 0.59 | 1.18 | 1.59 | 3.06 | 1.85 | 1.11 | 0.79 | 0.39 | 0.22 | 0.11 | 0.07 | 0.02 |
| **40-44** | 0.41 | 0.81 | 1.02 | 0.77 | 0.78 | 1.11 | 1.50 | 1.74 | 2.91 | 1.49 | 0.98 | 0.36 | 0.19 | 0.09 | 0.06 | 0.03 |
| **45-49** | 0.15 | 0.36 | 0.58 | 1.02 | 0.87 | 0.91 | 1.10 | 1.27 | 1.41 | 2.17 | 1.00 | 0.43 | 0.15 | 0.06 | 0.06 | 0.06 |
| **50-54** | 0.23 | 0.26 | 0.51 | 0.83 | 1.06 | 1.31 | 1.15 | 1.10 | 1.50 | 1.61 | 2.03 | 0.78 | 0.23 | 0.08 | 0.06 | 0.08 |
| **55-59** | 0.33 | 0.34 | 0.30 | 0.47 | 0.69 | 1.18 | 1.28 | 0.90 | 1.03 | 0.90 | 1.05 | 1.42 | 0.39 | 0.14 | 0.06 | 0.06 |
| **60-64** | 0.31 | 0.28 | 0.21 | 0.22 | 0.35 | 0.63 | 0.82 | 0.75 | 0.63 | 0.52 | 0.47 | 0.55 | 0.79 | 0.21 | 0.11 | 0.04 |
| **65-69** | 0.23 | 0.33 | 0.28 | 0.16 | 0.19 | 0.29 | 0.52 | 0.51 | 0.45 | 0.22 | 0.24 | 0.29 | 0.28 | 0.68 | 0.14 | 0.05 |
| **70-74** | 0.12 | 0.33 | 0.35 | 0.30 | 0.13 | 0.25 | 0.28 | 0.45 | 0.54 | 0.37 | 0.27 | 0.19 | 0.32 | 0.32 | 0.60 | 0.14 |
| **75+** | 0.21 | 0.25 | 0.41 | 0.32 | 0.12 | 0.14 | 0.24 | 0.32 | 0.37 | 0.42 | 0.42 | 0.22 | 0.13 | 0.17 | 0.15 | 0.30 |

(d)

| | 0-4 | 5-9 | 10-14 | 15-19 | 20-24 | 25-29 | 30-34 | 35-39 | 40-44 | 45-49 | 50-54 | 55-59 | 60-64 | 65-69 | 70-74 | 75+ |
|---|---|---|---|---|---|---|---|---|---|---|---|---|---|---|---|---|
| **0-4** | 1.16 | 0.62 | 0.32 | 0.16 | 0.23 | 0.46 | 0.84 | 0.88 | 0.54 | 0.18 | 0.18 | 0.13 | 0.09 | 0.06 | 0.04 | 0.03 |
| **5-9** | 0.44 | 1.65 | 0.69 | 0.22 | 0.11 | 0.31 | 0.68 | 0.85 | 0.87 | 0.29 | 0.13 | 0.11 | 0.07 | 0.05 | 0.03 | 0.02 |
| **10-14** | 0.27 | 0.96 | 3.38 | 0.74 | 0.25 | 0.20 | 0.41 | 0.81 | 1.22 | 0.59 | 0.30 | 0.11 | 0.05 | 0.06 | 0.05 | 0.04 |
| **15-19** | 0.14 | 0.32 | 1.20 | 4.16 | 1.17 | 0.56 | 0.45 | 0.68 | 1.08 | 1.19 | 0.67 | 0.19 | 0.07 | 0.04 | 0.03 | 0.02 |
| **20-24** | 0.15 | 0.14 | 0.21 | 1.37 | 3.16 | 1.48 | 1.01 | 0.90 | 0.82 | 1.08 | 0.79 | 0.39 | 0.12 | 0.03 | 0.03 | 0.03 |
| **25-29** | 0.41 | 0.18 | 0.11 | 0.50 | 1.46 | 3.22 | 1.63 | 1.19 | 1.02 | 0.86 | 0.92 | 0.54 | 0.21 | 0.05 | 0.02 | 0.02 |
| **30-34** | 0.69 | 0.66 | 0.39 | 0.27 | 0.80 | 1.55 | 3.11 | 1.69 | 1.25 | 0.92 | 0.71 | 0.53 | 0.28 | 0.08 | 0.04 | 0.03 |
| **35-39** | 0.70 | 0.95 | 0.75 | 0.52 | 0.59 | 1.18 | 1.59 | 3.06 | 1.85 | 1.11 | 0.79 | 0.39 | 0.22 | 0.11 | 0.07 | 0.02 |
| **40-44** | 0.41 | 0.81 | 1.02 | 0.77 | 0.78 | 1.11 | 1.50 | 1.74 | 2.91 | 1.49 | 0.98 | 0.36 | 0.19 | 0.09 | 0.06 | 0.03 |
| **45-49** | 0.15 | 0.36 | 0.58 | 1.02 | 0.87 | 0.91 | 1.10 | 1.27 | 1.41 | 2.17 | 1.00 | 0.43 | 0.15 | 0.06 | 0.06 | 0.06 |
| **50-54** | 0.23 | 0.26 | 0.51 | 0.83 | 1.06 | 1.31 | 1.15 | 1.10 | 1.50 | 1.61 | 2.03 | 0.78 | 0.23 | 0.08 | 0.06 | 0.08 |
| **55-59** | 0.33 | 0.34 | 0.30 | 0.47 | 0.69 | 1.18 | 1.28 | 0.90 | 1.03 | 0.90 | 1.05 | 1.42 | 0.39 | 0.14 | 0.06 | 0.06 |
| **60-64** | 0.31 | 0.28 | 0.21 | 0.22 | 0.35 | 0.63 | 0.82 | 0.75 | 0.63 | 0.52 | 0.47 | 0.55 | 0.79 | 0.21 | 0.11 | 0.04 |
| **65-69** | 0.23 | 0.33 | 0.28 | 0.16 | 0.19 | 0.29 | 0.52 | 0.51 | 0.45 | 0.22 | 0.24 | 0.29 | 0.28 | 0.68 | 0.14 | 0.05 |
| **70-74** | 0.12 | 0.33 | 0.35 | 0.30 | 0.13 | 0.25 | 0.28 | 0.45 | 0.54 | 0.37 | 0.27 | 0.19 | 0.32 | 0.32 | 0.60 | 0.14 |
| **75+** | 0.21 | 0.25 | 0.41 | 0.32 | 0.12 | 0.14 | 0.24 | 0.32 | 0.37 | 0.42 | 0.42 | 0.22 | 0.13 | 0.17 | 0.15 | 0.30 |

(e)

| | 0-4 | 5-9 | 10-14 | 15-19 | 20-24 | 25-29 | 30-34 | 35-39 | 40-44 | 45-49 | 50-54 | 55-59 | 60-64 | 65-69 | 70-74 | 75+ |
|---|---|---|---|---|---|---|---|---|---|---|---|---|---|---|---|---|
| **0-4** | 1.04 | 0.57 | 0.29 | 0.13 | 0.18 | 0.38 | 0.73 | 0.78 | 0.47 | 0.14 | 0.12 | 0.08 | 0.06 | 0.04 | 0.02 | 0.02 |
| **5-9** | 0.38 | 1.42 | 0.59 | 0.19 | 0.09 | 0.25 | 0.59 | 0.75 | 0.78 | 0.25 | 0.10 | 0.08 | 0.05 | 0.03 | 0.02 | 0.02 |
| **10-14** | 0.25 | 0.83 | 2.80 | 0.64 | 0.18 | 0.14 | 0.32 | 0.71 | 1.09 | 0.51 | 0.24 | 0.08 | 0.04 | 0.04 | 0.04 | 0.03 |
| **15-19** | 0.12 | 0.27 | 0.92 | 3.22 | 0.95 | 0.44 | 0.36 | 0.57 | 0.96 | 1.09 | 0.62 | 0.17 | 0.05 | 0.03 | 0.02 | 0.02 |
| **20-24** | 0.13 | 0.12 | 0.16 | 1.02 | 2.61 | 1.23 | 0.83 | 0.78 | 0.72 | 0.97 | 0.72 | 0.35 | 0.10 | 0.02 | 0.02 | 0.02 |
| **25-29** | 0.37 | 0.17 | 0.09 | 0.41 | 1.20 | 2.85 | 1.37 | 1.02 | 0.90 | 0.76 | 0.83 | 0.49 | 0.19 | 0.03 | 0.01 | 0.02 |
| **30-34** | 0.65 | 0.63 | 0.33 | 0.21 | 0.66 | 1.31 | 2.74 | 1.44 | 1.07 | 0.80 | 0.57 | 0.44 | 0.24 | 0.05 | 0.03 | 0.02 |
| **35-39** | 0.66 | 0.90 | 0.70 | 0.49 | 0.50 | 1.00 | 1.32 | 2.74 | 1.61 | 0.96 | 0.68 | 0.32 | 0.17 | 0.07 | 0.04 | 0.02 |
| **40-44** | 0.38 | 0.77 | 0.94 | 0.71 | 0.68 | 0.97 | 1.27 | 1.49 | 2.62 | 1.32 | 0.86 | 0.31 | 0.15 | 0.06 | 0.04 | 0.02 |
| **45-49** | 0.15 | 0.35 | 0.55 | 0.96 | 0.80 | 0.81 | 0.95 | 1.10 | 1.24 | 2.00 | 0.86 | 0.38 | 0.11 | 0.03 | 0.03 | 0.05 |
| **50-54** | 0.22 | 0.23 | 0.46 | 0.76 | 0.94 | 1.12 | 0.99 | 0.95 | 1.31 | 1.41 | 1.87 | 0.66 | 0.17 | 0.04 | 0.04 | 0.06 |
| **55-59** | 0.31 | 0.33 | 0.27 | 0.44 | 0.61 | 1.02 | 1.09 | 0.75 | 0.87 | 0.79 | 0.88 | 1.28 | 0.31 | 0.09 | 0.03 | 0.05 |
| **60-64** | 0.30 | 0.26 | 0.19 | 0.19 | 0.29 | 0.54 | 0.71 | 0.61 | 0.50 | 0.42 | 0.38 | 0.45 | 0.72 | 0.16 | 0.07 | 0.03 |
| **65-69** | 0.22 | 0.31 | 0.27 | 0.15 | 0.14 | 0.21 | 0.41 | 0.41 | 0.34 | 0.14 | 0.15 | 0.20 | 0.21 | 0.62 | 0.10 | 0.03 |
| **70-74** | 0.11 | 0.32 | 0.33 | 0.26 | 0.09 | 0.19 | 0.20 | 0.35 | 0.41 | 0.27 | 0.19 | 0.12 | 0.22 | 0.22 | 0.51 | 0.11 |
| **75+** | 0.21 | 0.25 | 0.39 | 0.31 | 0.10 | 0.11 | 0.18 | 0.27 | 0.32 | 0.36 | 0.37 | 0.18 | 0.10 | 0.12 | 0.11 | 0.27 |

(f)

**Figure S5.** Scenario specific contact matrices: (a) school closed and no social distancing, (b) school open and no social distancing, (c) school closed and weak social distancing, (d) school closed and weak social distancing+, (e) school closed and medium social distancing, and (f) school closed and strong social distancing.

# Supplementary Section C.

## The Effective Reproduction Number $R_t$.

The system has the disease-free state $x_0 = (S_i, 0,0,0,0)$. Let $x = (E_i, I_i, H_i)^{\mathrm{T}}$ for $i = 1,2,..,16$. $F(x)$ represents all of the new infections. The net transition rates of the corresponding compartments are represented by $V(x)$.

$$F(x) = \begin{pmatrix} \Lambda_i S_i \\ 0 \\ 0 \end{pmatrix}$$

where $\Lambda_i = \sum_{j=1}^{16} b_i m_{ij} \frac{I_j}{N_j}$.

$$V(x) = \begin{pmatrix} \alpha E_i \\ -\alpha E_i + q I_i \\ -q I_i + \gamma H_i \end{pmatrix}.$$

Thus, F and V are $48 * 48$ matrices at $x_0$ given by

$$F(x) = \begin{bmatrix} 0_{16,16} & A & 0_{16,16} \\ 0_{16,16} & 0_{16,16} & 0_{16,16} \\ 0_{16,16} & 0_{16,16} & 0_{16,16} \end{bmatrix}$$

where $A = diag\{b_1 S_1, b_2 S_2, \ldots, b_{16} S_{16}\}_{16} * M * diag\left\{\frac{1}{N_1}, \frac{1}{N_2}, \ldots, \frac{1}{N_{16}}\right\}_{16}$ and $M$ is the contact matrix.

$$\mathrm{V}(x) = \begin{bmatrix} B & 0_{16,16} & 0_{16,16} \\ -B & C & 0_{16,16} \\ 0_{16,16} & -C & D \end{bmatrix}$$

where $B = \alpha * I_{16}$, $C = q * I_{16}$, and $D = \gamma * I_{16}$.

Then, the inverse matrix of V is

$$\mathrm{V}^{-1} = \begin{bmatrix} B^{-1} & 0_{16,16} & 0_{16,16} \\ C^{-1} & C^{-1} & 0_{16,16} \\ D^{-1} & D^{-1} & D^{-1} \end{bmatrix}.$$

Hence, one can obtain the next generation matrix $G$ as

$$G = F\mathrm{V}^{-1} = \frac{1}{\mathrm{q}} * \begin{bmatrix} A & A & 0_{16,16} \\ 0_{16,16} & 0_{16,16} & 0_{16,16} \\ 0_{16,16} & 0_{16,16} & 0_{16,16} \end{bmatrix}.$$

Finally, the effective reproduction number $R_t$ is computed as the spectral radius $\rho(G)$ of the next generation matrix $G$, $i.e. R_t = \rho(G)$.

$$R_t = \rho(G) = \frac{\rho(A)}{q}.$$

# Supplementary Section D.

## Result Figures.

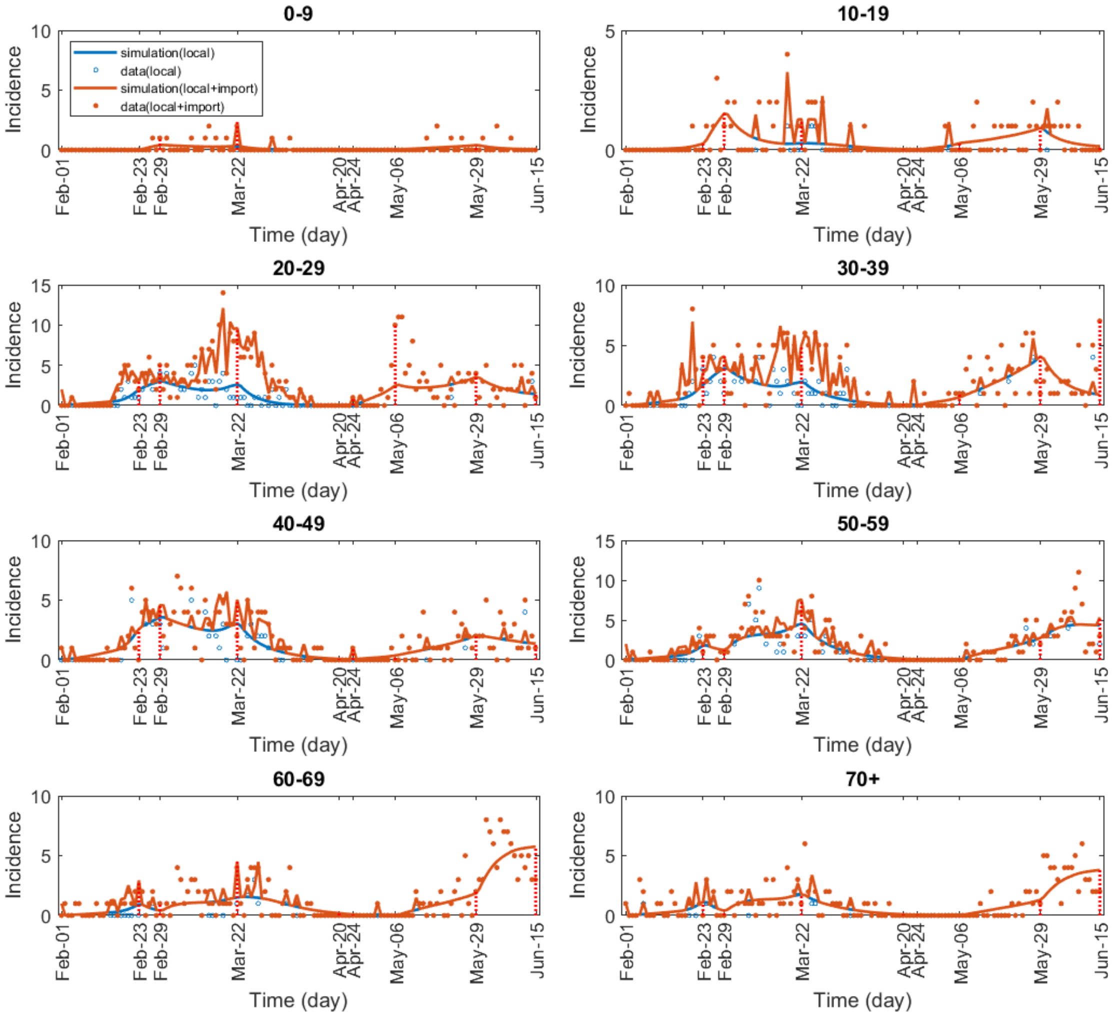


**Figure S6.** Estimation of transmission rate: Incidence of each age groups. Incidences by local transmission (local and imported transmission) are blue-colored (red-colored).

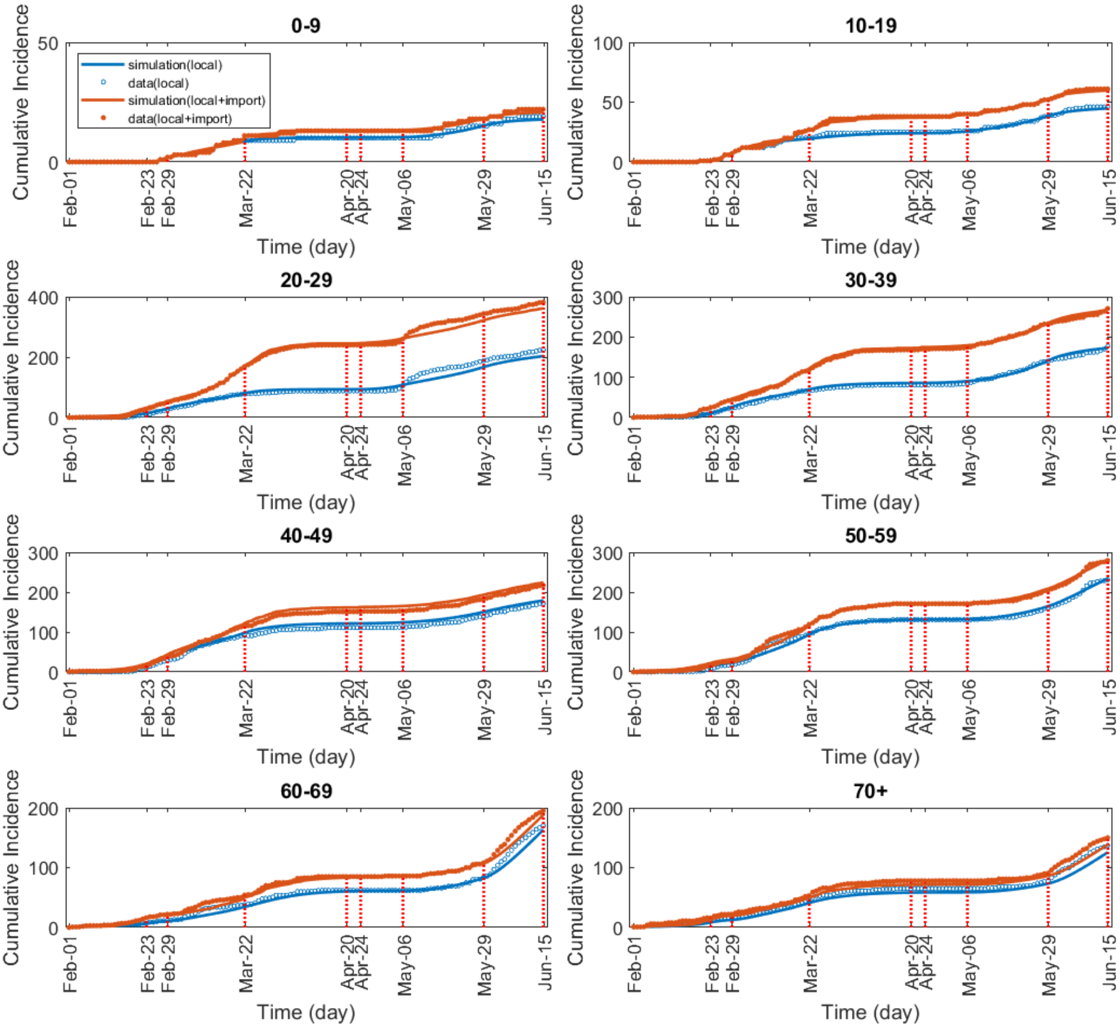


**Figure S7.** Estimation of transmission rate: Cumulative Incidence of each age groups. Incidences by local transmission (local and imported transmission) are blue-colored (red-colored).

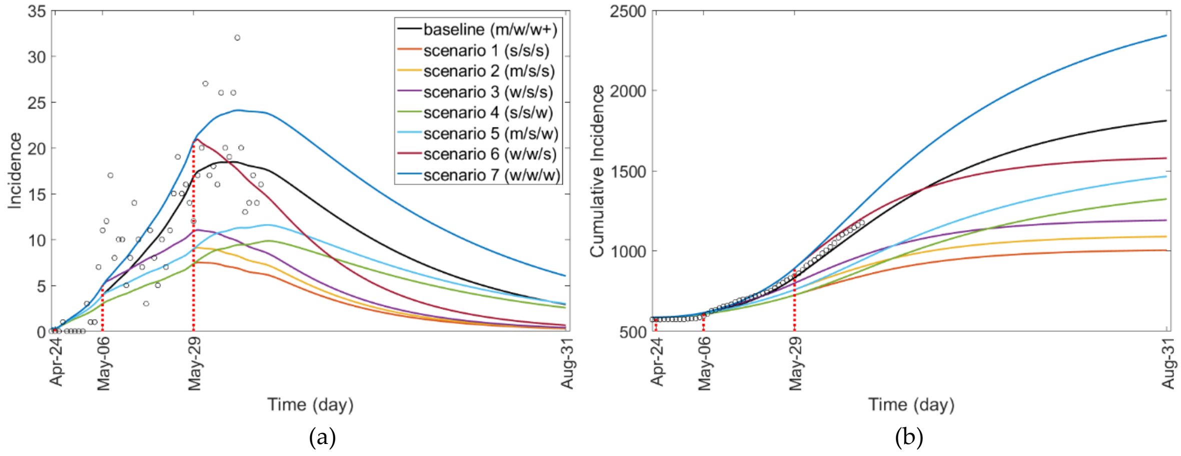


**Figure S8.** Scenario simulation: (a) Incidence and (b) Cumulative Incidence of all ages. s, m, w denote strong, medium, weak social distancing, respectively, and w+ denotes weak social distancing+. Black circles are actual incidence data.

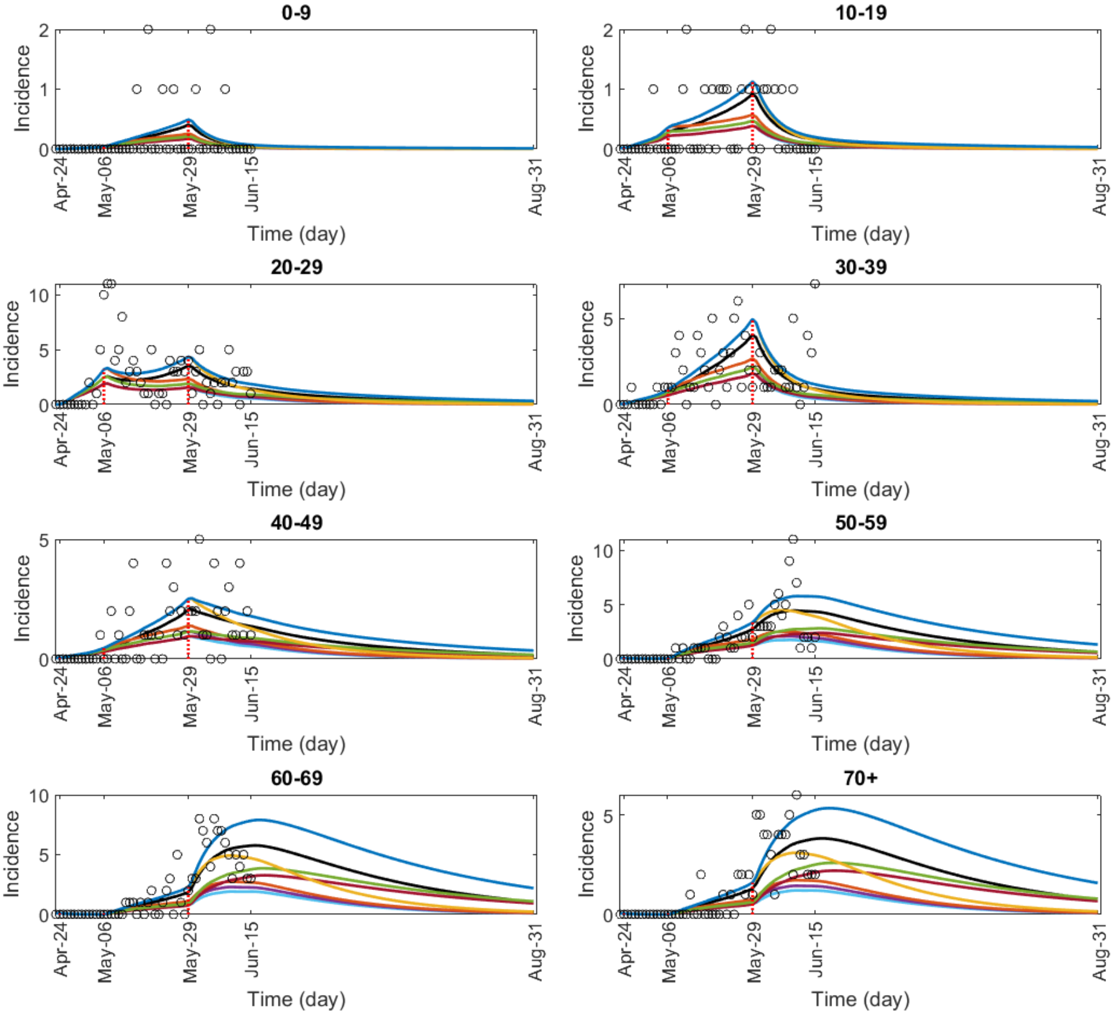


**Figure S9.** Scenario simulation: Incidence of each age groups.

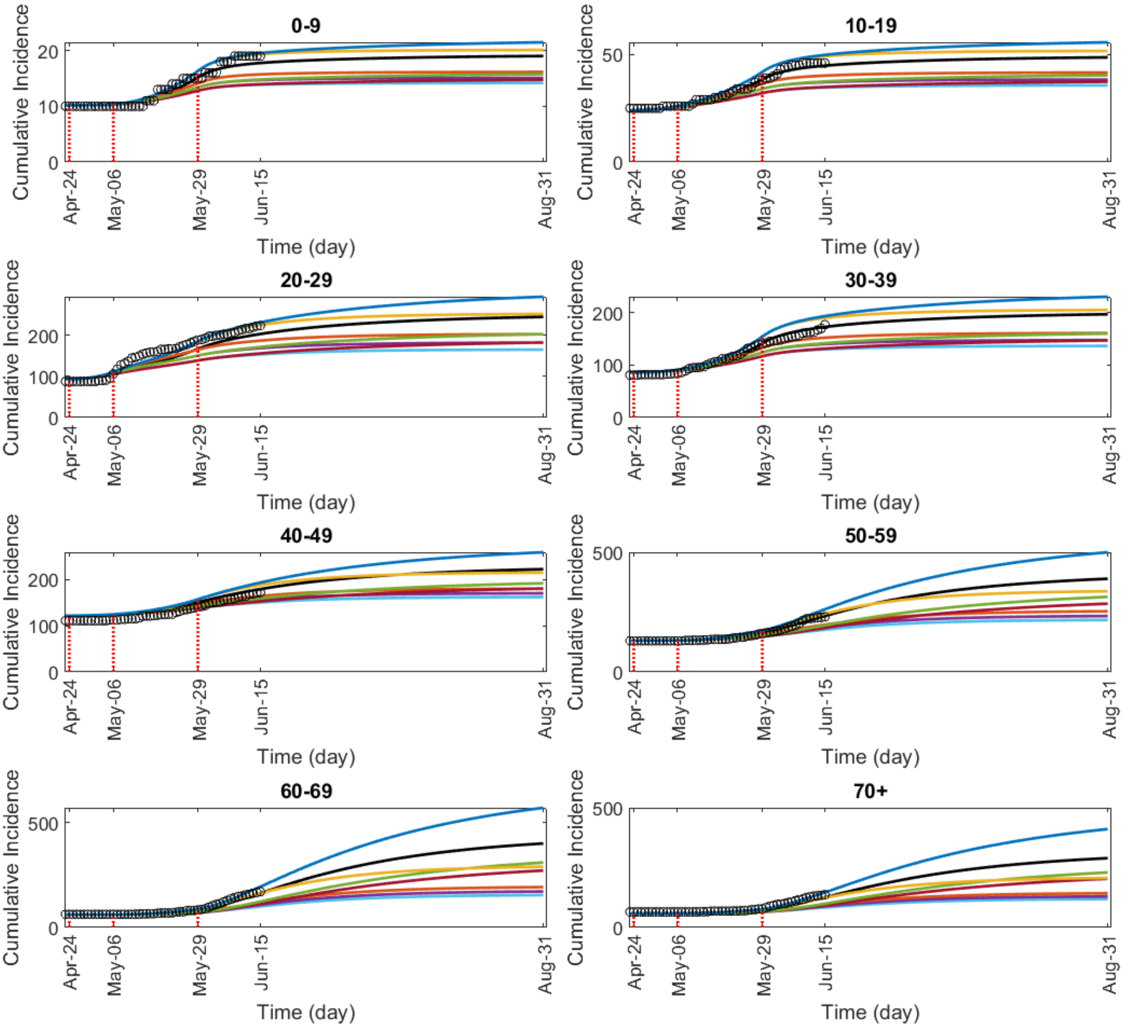


**Figure S10.** Scenario simulation: Cumulative Incidence of each age groups.

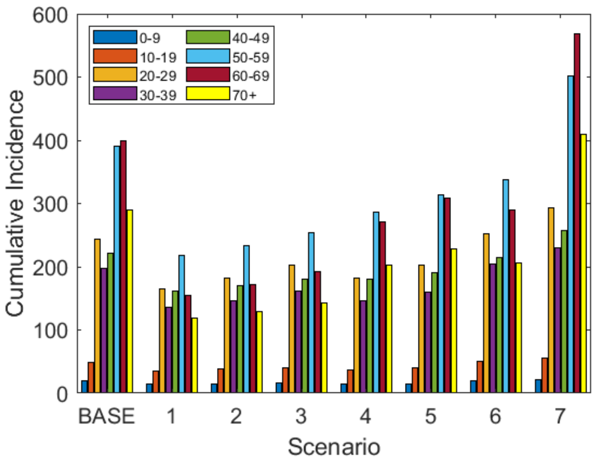


(a)

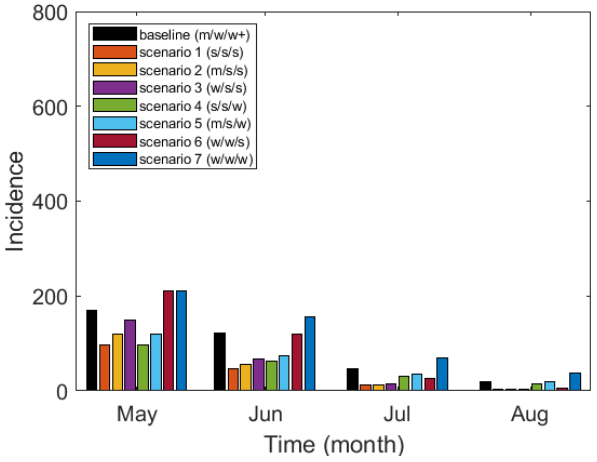


(b)

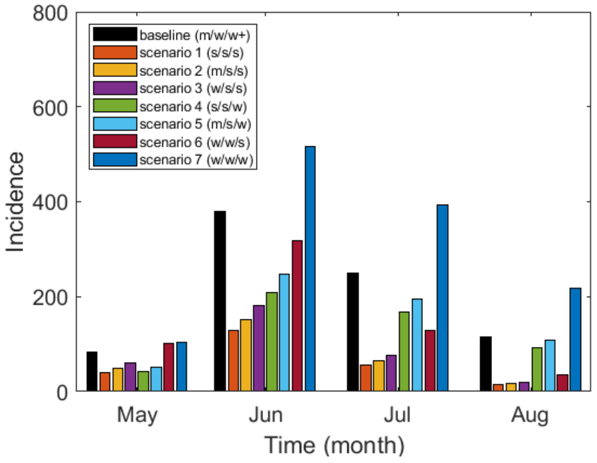


(c)

**Figure S11.** Other figures. (a) is scenario specific analysis on 31 Aug. 2020., (b) is 20-49 and (c) is 50 or older age group. s is strong-, m is medium-, w is weak- and w+ is weak social distancing+.

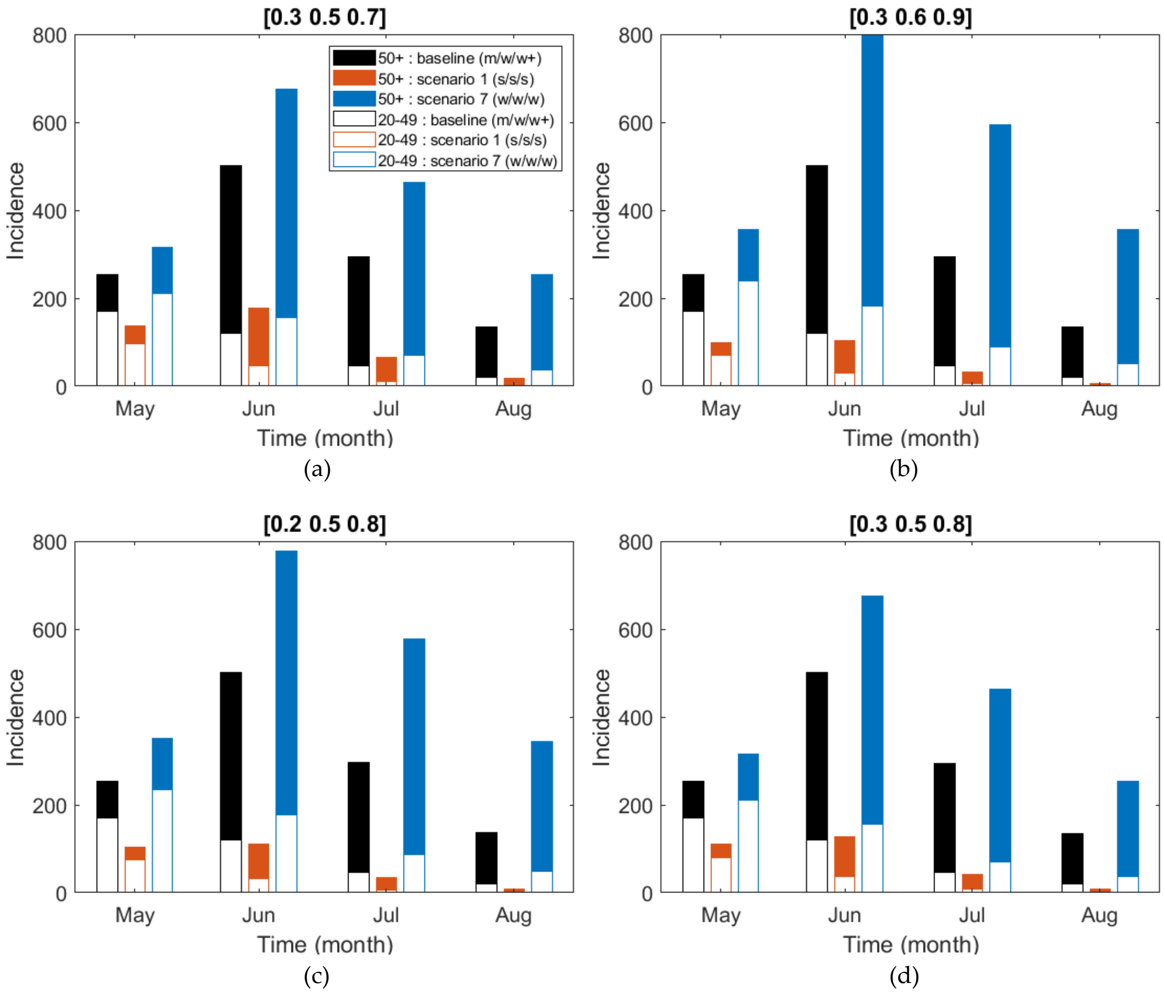


**Figure S12.** Monthly incidence of two age groups of 20-49 and 50 or older (50+) for the baseline, scenario 1 and 7 on different types $c_O$ levels for strong, medium and weak social distancing: (a) $c_O$ = 0.3, 0.6, 0.9, (b) $c_O$ = 0.3, 0.6, 0.9, (c) $c_O$ = 0.2, 0.5, 0.8, (d) $c_O$ = 0.3, 0.5, 0.8.

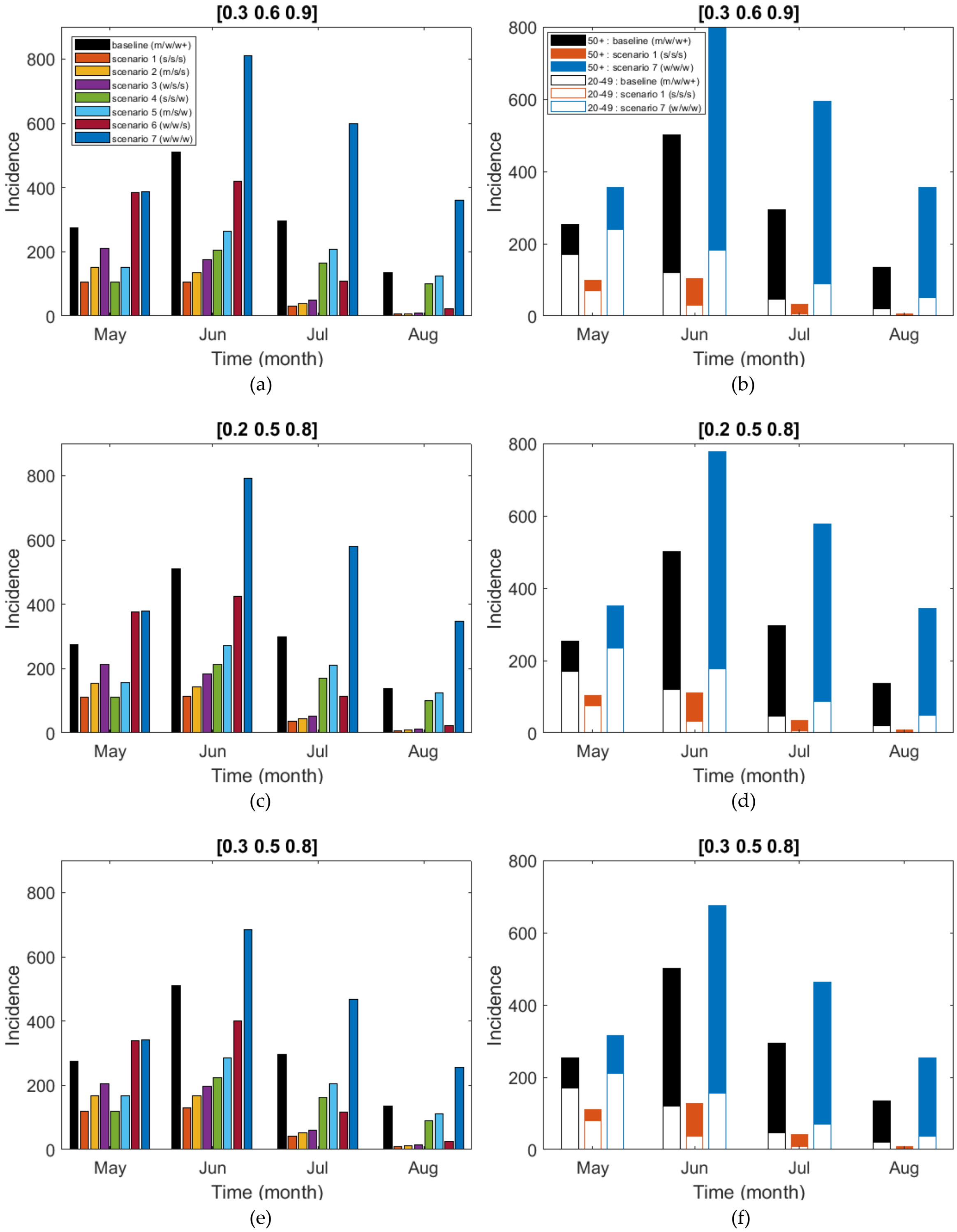


**Figure S13.** Monthly incidence of two age groups of 20-49 and 50 or older (50+) on different three types $c_O$ levels for strong, medium and weak social distancing: (a),(b) $c_O = 0.3, 0.6, 0.9$, (c),(d) $c_O = 0.2, 0.5, 0.8$, (e),(f) $c_O = 0.3, 0.5, 0.8$.
